# TPL2 Promotes Gastric Cancer Progression and Chemoresistance Through a Hypoxia-Induced Positive Feedback Loop with PPARδ

**DOI:** 10.7150/ijbs.111998

**Published:** 2025-09-12

**Authors:** Keng-Li Lan, De-Wei Lai, Cheng-Ning Yang, Hung-Chuan Pan, Hui-Ting Ou, Szu-I Yu, Tsung-Che Hsieh, Yi-Lun Ye, Chia-Yang Chan, Kin-Long Chou, Sheng-Mao Wu, Li-Wei Shen, Chin-Chang Shen, Lujen Chen, Shing-Hwa Liu, Chien‑Shan Chiu, Jack L Arbiser, Meei-Ling Sheu

**Affiliations:** 1 Institute of Traditional Medicine, College of Medicine, National Yang-Ming Chiao-Tung University, Taipei, Taiwan.; 2 Department of Heavy Particles & Radiation Oncology, Taipei veterans General Hospital, Taipei, Taiwan.; 3 Department of Medical Research, Taichung Veterans General Hospital, Taichung, Taiwan.; 4 Department of Dentistry, School of Dentistry, College of Medicine, National Taiwan University, Taipei, Taiwan.; 5 Department of Neurosurgery, Taichung Veterans General Hospital, Taichung, Taiwan.; 6 Ph.D. Program in Translational Medicine, Rong Hsing Research Center for Translational Medicine, Doctoral program in Biotechnology Industrial Management and Innovation, Biomedical Research and Industrialization Center, National Chung Hsing University, Taichung, Taiwan.; 7 Institute of Biomedical Sciences, National Chung Hsing University, Taichung, Taiwan.; 8 Department of Medical Research, Tungs' Taichung MetroHarbor Hospital, Taichung, Taiwan.; 9 Institute of Nuclear Energy Research, Atomic Energy Council, Taoyuan, Taiwan.; 10 Department of Pathology and Laboratory Medicine, Taichung Veterans General Hospital, Taichung, Taiwan.; 11 Institute of Toxicology, College of Medicine, National Taiwan University, Taipei, Taiwan.; 12 Department of Medical Research, China Medical University Hospital, China Medical University, Taichung, Taiwan.; 13 Department of Dermatology, Taichung Veterans General Hospital, Taichung, Taiwan.; 14 Department of Dermatology, Emory University School of Medicine, Winship Cancer Institute, Atlanta Veterans Administration Health Center, Atlanta, Georgia, USA.; 15 These authors contributed equally to first author in this work.; 16 The author contributed equally to corresponding author.

**Keywords:** TPL2, PPARδ, hypoxia, proliferation, chemoresistance

## Abstract

Tumor progression locus 2 (TPL2), a member of the MAP3K serine/threonine protein kinase family, is implicated in immune responses and pro-inflammatory protein phosphorylation. Emerging evidence suggests its role in tumorigenesis; however, its contribution to gastric cancer (GC) development remains unclear. Patients' disease progression and tumor tissues obtained were used to perform gene expression and GSEA analysis. Immunohistochemical staining, *EMSA*, ChIP, immunoprecipitation analyses, confocal microscope image and molecular docking were conducted to investigate the relationship between TPL2 and Peroxisome proliferator-activated receptor delta (PPARδ). Xenograft mouse models were used to study the role of PPARδ/TPL2 axis in tumor growth and the efficacy of blocked TPL2. TPL2 expression was significantly upregulated in GC tissues compared to adjacent normal tissues, and high TPL2 levels correlated with poor patient outcomes. Silencing TPL2 via siRNA or pharmacological inhibition suppressed GC cell proliferation and enhanced sensitivity to doxorubicin (Adriamycin), whereas TPL2 overexpression promoted tumor growth and chemoresistance. Mechanistically, TPL2 activated hypoxia/PPARδ signaling by interacting with PPARδ, thereby enhancing its transcriptional activity. Furthermore, PPARδ transcriptionally upregulated TPL2 expression, establishing a positive feedback loop. Functional studies confirmed the interdependent relationship between TPL2 and PPARδ in regulating GC cell proliferation and drug resistance. This study identifies a novel TPL2/PPARδ positive feedback regulatory loop that drives GC progression and chemoresistance. Targeting this axis may provide new therapeutic strategies not only for GC but also for other diseases associated with pathological hypoxia.

## Introduction

Gastric cancer (GC) has been indicated as the second leading cause of cancer deaths in the world malignancies, and it is also high morbidity and mortality rate; particularly prevails in East Asia 1. GC incidence and mortality are highly variable by region and highly dependent on diet and *H*.* Pylori infection*, emphasizing the urgency and importance of deeply exploring its initiation and progression mechanisms 2. There are numerous molecules involved in the pathogenesis of GC, among which, environmental agent, infections and dietary *factors* those are participate in the most important ones 3. Multiple inflammation cascade pathways have been implicated in the transformation of chronic epithelium inflammation to GC, including the COX-2/PGE_2_, IL-8/NF-κB, IL-1β/TAB1, TNFα/VEGF, IL-6/STAT1, and MCP-*1*, pathways 1-3. Overarching dysregulation factor encompasses the interrelatedness, in these complicated signaling cascade may cause to aberrant biological behaviors in stomach tissue, such as tumor cell transformation, inhibited apoptosis, promoted cell proliferation, strengthen metastasis, invasive and enhanced drug resistance, angiogenesis and immune escape 4. Noteworthy, tumor-hypoxia enhances chemoresistance of cancer cells and correlated with poor outcome for patients. The query of how these molecules and signaling cascade are evoked and maintained in GC is still enigma. The first priority to investigate how these factors interaction and signaling pathways and mutual regulation.

Oncogenic kinase Tumor Progression Locus 2 (TPL2) is a serine/threonine protein kinase that consistent of a C-terminal domain containing a degron sequence (a.a. 435-457) that control its stability and specific activity.^5^-^7^ The N-terminal extension consists of an α-helix (αN2) and a short, parallel β-sheet (βN1 and βN2), a flexible 15-amino acid P-loop extension, a shortened C helix relative to canonical kinase domain folds 7-9. Interestingly, reports shown TPL-2, ABIN-2 stoichiometric complex with NFκB1 p105 and is essential for TPL-2 protein stability 10. In addition, IκB kinase-induced interaction of TPL-2 kinase with 14-3-3 is essential for Toll-like receptor activation of ERK-1 and -2 MAP kinases 11. Other members demonstrated TPL2 may also operate in the nucleus as a physical and functional partner of nucleophosmin (NPM/B23), a major nucleolar phosphoprotein with diverse cellular activities linked to malignancy 12. TPL2, are known to participate in macrophages, which involved in the cross talk between adipocytes and macrophages that promotes inflammatory changes and alteration of insulin signaling in adipocytes 13. In cancer, TPL2 seems to have ambivalent and inconclusive roles by being able to act as a tumor suppressor or as a driver of tumorigenesis. TPL2 knock-out background has been shown TPL2 kinase take as a suppressor of lung carcinogenesis 14. Moreover, TPL2 promotes p53-mediated tumor suppression in lung cells through a JNK-NPM pathway 14-16, and beneficial in prostate cancer and breast cancer 9, 16, 17. Besides, TPL2 in CD40 and TNFR-transduced signals that result in ERK activation and Ig class switching. CD40 recruits both TPL2 and TRAF6 in a complex suggesting that plays a critical role in the transduction of signals that activate ERK, subsequently 18. Following our previous study demonstrated that inhibition of TPL2 improved diabetic vasculopathy through the blockage of the inflammasome complex 19, 20.

The highly elevated TPL2 has been found to be a potential oncogenic factor in verity types of human cancers. Studies have done through pancreatic cancer to colon cancer 19-25. However, the expression profile, functional verification, and regulatory mechanism of TPL2 in chemoresistance of GC remain unclear. Herein, in the present study, we described the clinical pathological expression of TPL2 and immunohistochemical staining in GC clinical subject tissues 26-30. We elucidated its role in GC proliferation *in vitro* and *in vivo* models for analysis of resistance. More importantly, we set up a link between TPL2 and hypoxia/PPARδ signaling, proposing TPL2 could intensify this signaling cascade by a positive feedback loop to promote GC development and chemoresistance.

## Materials and Methods

Many of the methods listed here have been published previously but are repeated here for clarity 19, 20, 22-25, 31-34. The information for primary and secondary antibodies was listed in Supplementary Table 1 and Supplementary Table 2, respectively. The information for primer sequences used in qRT-PCR was listed in Supplementary Table 3. The information for luciferase reporter assays was listed in Supplementary Table 4.

### Cell culture

Cell culture systems were used as described previously gastric adenocarcinoma 19, 20, 22-25, 31-34. Human gastric cancer cell lines, AGS (moderately differentiated), NCI-*N87 cells* (highly differentiated and divide more slowly) and MKN45 or SCM-1 cells (poorly differentiated) were supplied by the cell bank of Taipei Veterans General Hospital (Taiwan). Cells were grown in RPMI medium supplemented with 10% FBS, 100 U/ml penicillin, and 100 mg/ml streptomycin (complete medium) at 37 °C in a humidified incubator with 5% CO_2_/95% air mixture under normoxic control. In hypoxia chamber for the hypoxia incubation was performed in a hypoxia chamber water jacket incubator (Astec Co., Kasuya, Fukuoka, Japan) humidified with 1% O_2_, 5% CO_2_, and 94% N_2_ at 37 °C. During experiments, cells were plated in six-well plates cultured with serum-free medium (starved medium) overnight and then treated with inhibitors drugs. In some experiments, transfection of cancer cells was performed using a Lipofectin reagent overnight and further treated with hypoxia. Induction of hypoxia was performed either by Western blot for target protein expression, luciferase assay, or MTS assay as indicated.

### Western blot analysis and immunoprecipitations (IP)

Immunoblotting was performed as described previously 19, 20, 22-25, 31-34. Proteins immunoprecipitated from total cell lysate (800 μg) and whole cell lysate proteins (80 μg) for 10% input were separated by SDS-PAGE and electrophoretically transferred to nitrocellulose membranes. After blocking, the blots were incubated with antibodies (Supplementary Table 1) overnight. Membranes were then incubated for 1 h with secondary antibody (Supplementary Table 2). Detection was performed by ECL (Amersham) and by chemiluminescence using Kodak X-Omat film. The antibodies used in this study were listed in Supplementary Table-1. Protein pre-clean (800 μg) was incubated with pre-immune serum for 1 h at 4ºC with gentle agitation. Supernatant further were incubated with specific antibodies and immobilized immunoprecipitated with protein A-Sepharose for overnight at 4ºC. Beads were pelleted by centrifugation at 2,500 × g, washed three times with IP buffer, and analyzed by electrophoresis and immunoblot as it was indicated above.

### Transfection

Cancer cells were transfected with 1 μM siRNA-TPL2, scramble-RNA (Santa Cruz Biotechnology); 5 μg/ml shRNA-TPL2 or shEGFP, or TPL2-overexpressed plasmid 1 mg/ml pcDNA-TPL2 (Genome Research Center, National Yang-Ming University) using Lipofectin reagent (Invitrogen) according to the manufacturer's instructions.

### MTS cell proliferation assay

Cell lines were seeded at a density of 3,000 cells per well in 96-well plates. Cells cultured in 96-well plates were mixed with CellTiter 96 Aqueous One Solution Reagent containing a tetrazolium compound [3-(4,5-dimethylthiazol-2-yl)-5-(3-carboxymethoxyphenyl)-2-(4-sulfophenyl)-2H-tetrazolium, inner salt; MTS] (Promega, Madison, WI). Proliferation was determined by MTS assay according to the instructions of the manufacturer by measuring the absorbance at 490 nm using a 96-well plate reader (Biotek).

### [^3^H] Thymidine incorporation assay

[^3^H] Thymidine incorporation assay was performed to assess inhibition of DNA synthesis induced by various induction as indicated. Cells (~5 × 10^3^ per well) were treated with required concentrations of the drug after seeding in 96-well plate. After 18 h incubation with the compounds, [^3^H] thymidine was added (0.2 μCi per well) and incubated for 6 h. The cells were washed with PBS; precipitated with 5% trichloroacetic acid and solubilized in 0.2 N NaOH. The relative cell viability was calculated as percentage thymidine incorporation over untreated control. Cells were harvested, and the radioactivity was measured and quantified as counts per minute (cpm) using a scintillation counter.

### Animal model

All animal care and experimental procedures were approved and conducted by the Committee for Animal Experiments of National Chung Hsing University (Approval Document NCHU-100-26). All analyses of the experiments were under blinded conditions. Nude mice were injected intraperitoneally with cancer cells SCM1 or MKN45 transfection shEGF and shTpl2 (5 μg/ml) or TPL2 inhibitor (2 mg/kg, twice/weekly, i.p.) for 1 month. The body organs were examined for tumor growth, and various tissues were processed for histological examination. The peritoneal dissemination assay was conducted as previously described. Transfections of shRNA into cancer cells were performed using Lipofectin. The cancer cells (1 x 10^6^) cells were transfected for 24 h and then each of the mice received one of the cell types via intraperitoneal injection to the abdominal cavity for 30 days. Quantification of the tumor mass was evaluated by one slide section and vessels were estimated by counting five randomly chosen high power fields.

### Soft agar colony formation assay

Cells (10000 cells/well) were suspended in 10% FBS-RPMI 1640 containing 0.7% agar. The cells were then placed into a 6-well culture plate containing a hard agar base composed of 10% FBS-RPMI 1640 and 1.4 % agar. The plates were incubated for 4 weeks. The cells were stained with 0.05% crystal violet overnight at 37 degrees. Colonies were visualized and counted by light microscopy at X20 magnification. The results shown are representative of at least five independent experiments.

### RNA isolation and qRT-PCR (quantitative real-time PCR)

Total RNA was extracted from CRC cells using TRizol regent (Sigma-Aldrich, Germany) according to the manufacturer's protocol. RNA (2 μg) was reverse-transcribed using a PrimeScript RT reagent kit (TaKaRa, Japan) (Supplementary Table 3). qRT-PCR was performed using TB Green® Premix Ex Taq™ kit (Takara, Japan) in an ABI 7500 PCR system (Applied Biosystems, USA). All the results were expressed at least four independent experiments and presented. The mRNA level of each sample was normalized to β-actin via 2^-ΔCt^ method.

### Luciferase reporter assay

Cells at 60% confluence were co-transfected with 0.2 μg of the promoter reporter construct peroxisome proliferator response element (PPRE), and 0.1 mg of a thymidinekinase promoter driven Renilla-luciferase vector (pRLTK; Promega, Mannheim, Germany). After incubation, cells were lysed and processed relative luciferase activities using the Dual Luciferase Kit (Promega) as described by the manufacturer. Luciferase activity was normalized to Renilla firefly activity for transfection efficiency and recorded by a luminometer (LKB, Rockville, MD). For the signaling pathway analysis, 8 luciferase reporter constructs, including AP-1-luc, ARE-luc, HRE-luc, NF-κB-luc, STAT3-luc, PPRE-luc, SMAD2/3-luc, WNT-luc, and represent eight signaling pathways (Cignal Reporter Assay Kits, QIAGEN) (Supplementary Table 4). These plasmids were respectively co-transfected with Renilla luciferase plasmid pRL-SV40 into with/without TPL2 knockdown cells and control cells in a 6-well plate, and measured the relative luciferase activities by dual-luciferase reporter assay kit as described above. All transfections were routinely performed in triplicate and the experiments were repeated three times.

### Immunohistochemistry analysis

Sections (4-5 μm thick) cut from 10% formalin-fixed, paraffin-embedded eye samples were used for hematoxylin-eosin staining. Immunohistochemistry analysis was performed as described previously. The commercial antibodies used in this study were listed together with their source in Supplementary Tables 1 and 2.

### Immunofluorescence and laser scanning confocal microscopy

Cells were prepared and the immunofluorescence was determined by laser scanning confocal microscopy (LSCM, TCS SL, Leica, Wetzlar, Germany) as previously described. Images were background-subtracted and merged using the Confocal Assistant MetaMorph software program, and processed with Adobe Photoshop software.

### Electrophoretic mobility shift assay

The EMSA was performed as described previously. The oligonucleotide with the PPARδ consensus binding sequence used was (5′-AGGTCA-3′); -389 to -358 human; The forward primer (5′-GGGGCGCGCGGAAAAAGGTCTGGTGACTGCCC-3′), whereas the reverse primer was (5′-GGGCAGTCACCAGACCTTTTTCCGCGCGCCCC-3′). DNA-protein complexes were resolved on 6% nondenaturing polyacrylamide gels and visualized by exposure to autoradiographic films.

### Chromatin immunoprecipitation assay

The assay protocol was modified as described previously A fragment (221 bp) of the TPL2 promoter containing putative PPARδ-binding sites was used. The forward primer was -496≈-477 (5′-TAGCAATCGGACCCACAGTC-3′), whereas the reverse primer was -295≈-276 (5′-GGGCGCGAGTACACTAAGAT-3′). Thirty cycles of PCR were conducted at 94°C for 30 seconds, at 64.8°C for 30 seconds, and at 72°C for 45 seconds. The PCR products were precipitated and run on a 1.5% agarose gel.

### Protein-protein docking

Protein docking simulation by ZDOCK, which performs a full rigid-body search of docking orientations between two proteins. The current version, 3.0.2, includes performance optimization and a novel pairwise statistical energy potential. The protein-protein docking of the TPL2: PPARδ complex used the human structures found in *RCSB* PDB, respectively. A series of docking runs was performed using the ZDOCK server, using a fast Fourier transform-based docking algorithm that takes into account pairwise shape complementarity, desolvation, electrostatics, and statistical potential. Select residues to block from the binding site at TPL2.pdb on 290 chain A THR and PPARδ.pdb on 256 chain B THR. ZDOCK results reliably indicated that the TPL2 associates with the PPARδ in an orientation. In terms of structural displays, unless otherwise indicated, structural figures presented here were prepared by PyMOL. We also used ClusPro web server for docking lowest energy and weighted score -1044.6~ -903.7.

### Association between TPL2 expression levels and patient survival

We utilized public web servers for gastric cancer and stomach adenocarcinoma databases to investigate TPL2 expression patterns across normal and tumor tissues, as well as overall survival probability. These platforms enable the future analysis and validation of newly identified gene expression-based biomarkers and signatures in both currently studied and unexplored patient subgroups. The analysis of target genes using GENT2 (http://gent2.appex.kr/gent2/) is presented to explore TPL2 expression patterns across normal and tumor tissues, encompassing tissue-wide gene expression patterns across 72 paired samples with accompanying statistical tests. Survival analysis was performed using Kaplan-Meier Plotter (https://kmplot.com/analysis/), which generated Kaplan-Meier survival curves for overall survival (OS), first progression (FP), and post-progression survival (PPS). The results are visualized as Kaplan-Meier curves, with user options for probe set selection (specific probe sets or all probe sets per gene) and the ability to exclude dataset GSE62254. (GSE62254 exhibits markedly different characteristics, including longer survival and shifted expression, suggesting its exclusion when analyzing all datasets together.) Additionally, UALCAN (https://ualcan.path.uab.edu/analysis.html), a portal designed to facilitate tumor subgroup gene expression and survival analyses based on sample type, tumor grade, and specific cancer subtypes, was employed for stomach adenocarcinoma data. Through systematic and integrated bioinformatics analysis, we identified potential key genes and their association with poor prognosis in gastric cancer.

### Statistical analyses

The data and statistical analysis comply with the recommendations and requirements of the British Journal of Pharmacology on experimental design and analysis in pharmacology. The study statistical analyses were performed with SAS software (SAS Institute, Cary, N.C.). The values were presented as mean ±SD. Analysis of variance, followed by Fisher's least significant difference test, was performed for all data. Statistical significance was set at p < 0.05.

## Results

### TPL2 expression is upregulated in GC patients

**Figure 1:**
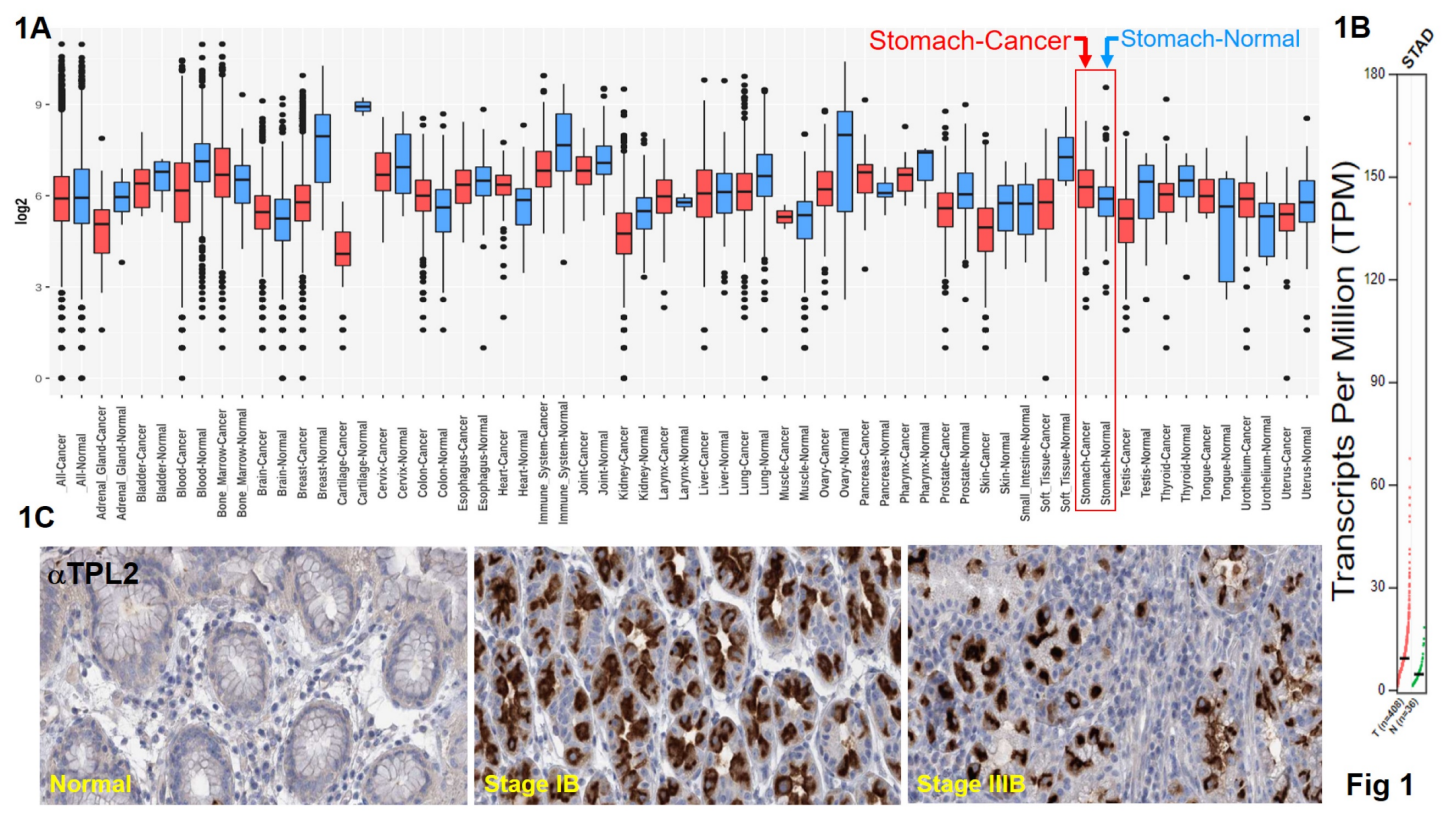
** TPL2 was overexpressed in GC and significantly associated with poor prognosis.** The public web server GENT2 is presented for exploring expression patterns across normal and tumor tissues. **1A.** TPL2 mRNA level was higher in gastric adenocarcinoma tissues than normal tissues from the GENT2, a platform for exploring Gene Expression patterns across Normal and Tumor tissues and search tissue-wide expression profile across various tissues. The Boxplot for gene expression profile across cancer experiments [GPL96 platform (HG-U133A)]. **1B.** Match TCGA normal data shown Transcripts Per Million (TPM) in gene expression profile, T (n=408), N (N=36). TPL2 was upregulated in tumor tissues compared with normal tissues in gastric cancer carcinoma samples. **1C.** Representative immunostaining of TPL2 expressions in human normal gastric mucosa (left), moderately differentiated intestinal type adenocarcinoma (middle), poorly differentiated intestinal type adenocarcinoma (right). Scale bar: 25 μm.

**Figure 2:**
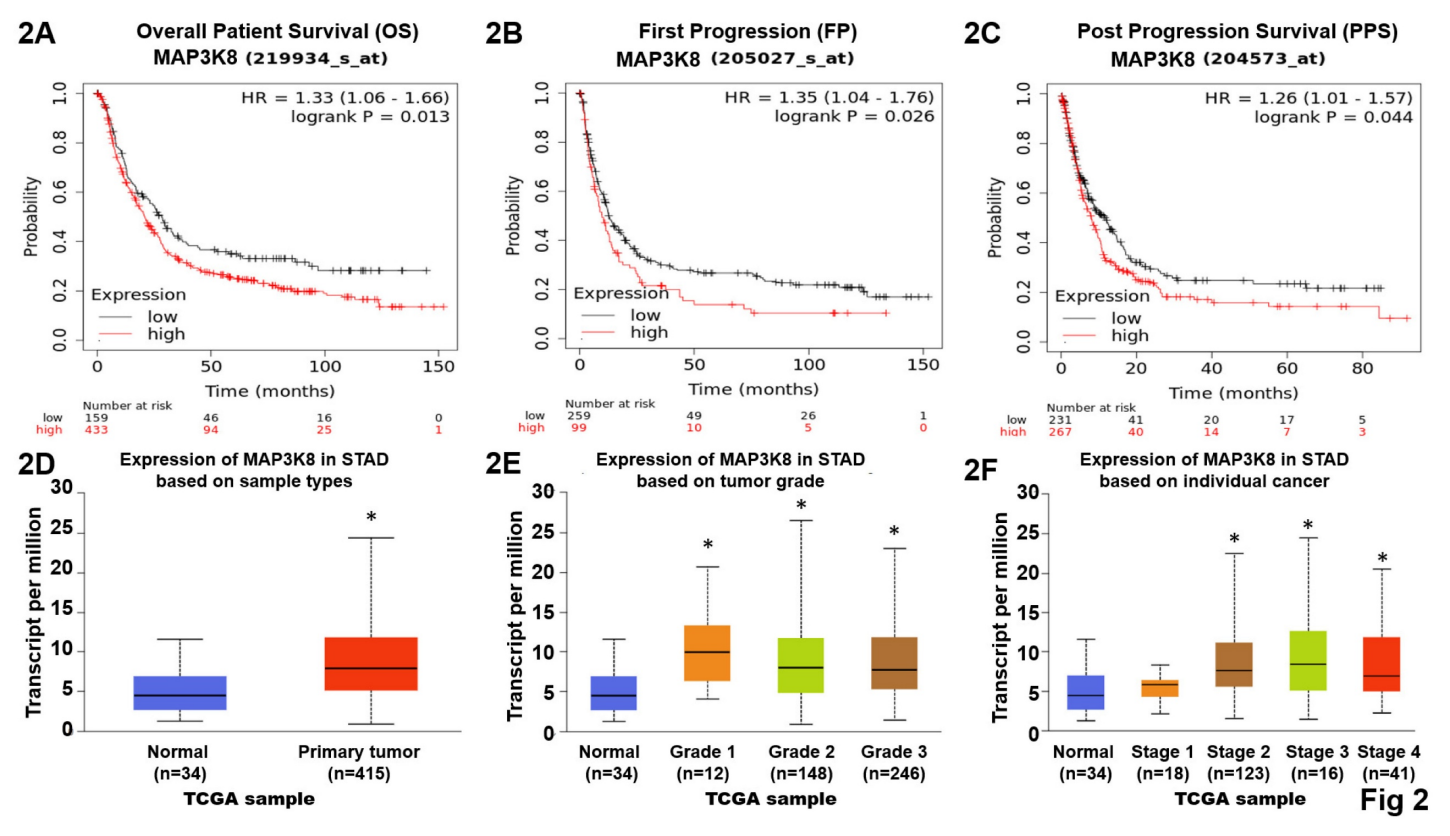
** Highly MAP3K8 mRNA expression decreased overall survival probability in GC patients.** These explores and presents data from the public web server KM Plotter for gastric cancer (https://kmplot.com/analysis/index.php?p=service&cancer=gastric) and the University of Alabama at Birmingham Cancer Data Analysis Portal (UALCAN) for stomach adenocarcinoma (https://ualcan.path.uab.edu/). **2A.** High TPL2 expressions were associated with decreased overall survival (OS) probability in gastric cancer. Data obtained were from the dataset (219934_s_at, *n* = 592) through a comprehensive search using Kaplan-Meier plotter.com for TPL2 evaluation. **2B.** Elevated TPL2 expressions were associated with decreased First Progression (FP) probability in gastric cancer. Data obtained were from the dataset (205027_s_at, *n* = 358) via Web-Based Survival Analysis using Kaplan-Meier plotter.com for TPL2 validation. **2C.** Raising of TPL2 expressions were associated with decreased Post Progression Survival (PPS) probability in gastric cancer. Data obtained were from the dataset (204573_s_at, *n* = 498) by database using Kaplan-Meier plotter.com for TPL2 analysis. Kaplan-Meyer plot of two groups of GC patients classified by TPL2 expression. Statistical analyses were performed with log-rank test as indicated. Red, high expression group; black, low expression group. **2D.** Expression of TPL2 in stomach cancer patients based on sample types. Normal (n=34), primary (n=415). * *p <* 0.05 compared to the Normal group. **2E.** Expression of TPL2 in stomach cancer patients based on tumor grade. Normal (n=34), grade1 (n=12), grade2 (n=148), grade3 (n=246). * *p <* 0.05 compared to the Normal group. **2F.** Expression of TPL2 in stomach cancer patients based on individual cancer stages. Normal (n=34), grade1 (n=18), grade2 (n=123), grade3 (n=169), grade4 (n=41). * *p <* 0.05 compared to the Normal group.

Data from the GENT2, a platform for exploring gene expression patterns across normal and tumor tissues and search tissue-wide expression profile across various tissues. The Boxplot for gene expression profile across cancer experiments [GPL96 platform (HG-U133A)]. We found that stomach cancer highly expression TPL2 compared with normal tissues (Fig. 1A). We further to match TCGA normal data shown Transcripts Per Million (TPM) in gene expression profile, T (n=408), N (N=36) (Fig. 1B). Subsequently, immunohistochemical analysis (IHC) was conducted to examine TPL2 protein expression in a GC real word tissue sample. According to the Cancer Institute of Tissue Bank of Taichung Veterans General Hospital (VGHTC) data collection, TPL2 protein expression was significantly higher in tumor tissue (n=40) than normal tissue (n=39) (Fig. 1C). These results consistent with those online database analyses regarding the protein expression of TPL2 was also significantly increased in GC tissues Fig. 2 (Fig. 2A, Overall Patient Survival (OS); Fig. 2B, First Progression (FP); Fig. 2C, Post Progression Survival (PPS)). Furthermore, we evaluated the relationship between TPL2 expression and patients' prognosis. Expression of TPL2 in stomach cancer patients based on sample types (Fig. 2D), tumor grade (Fig. 2E) and individual cancer stages (Fig. 2F). The results showed that high expression of TPL2 was correlated with decreased overall survival and associated with tumor progression. These data strongly suggested that TPL2 may play important roles in GC.

### TPL2 knockdown or TPL2 inhibitor restrains GC cell proliferation in vitro and tumorigenicity in vivo

**Figure 3:**
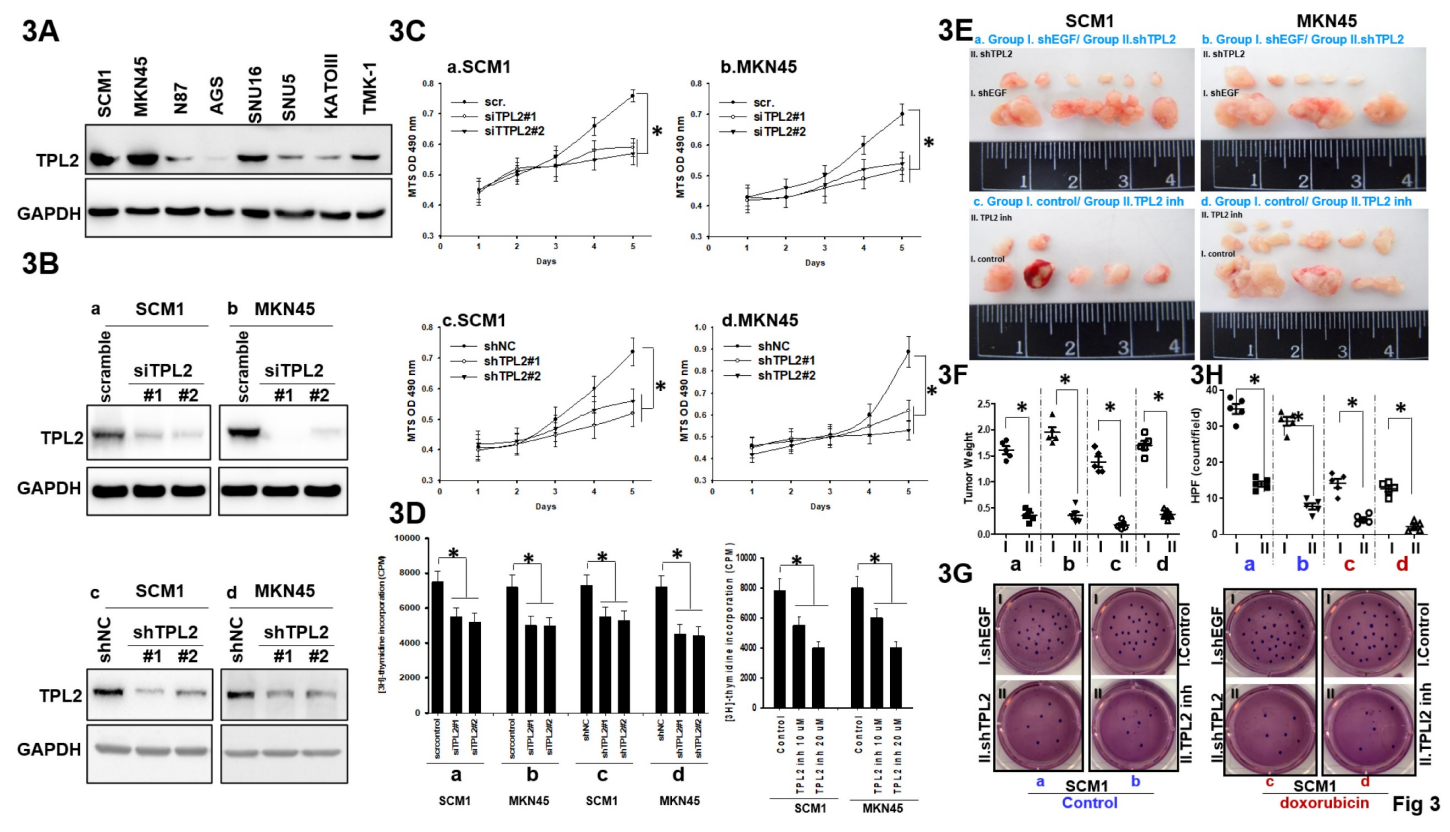
** TPL2 downregulation suppressed GC cell proliferation, tumorigenecity and doxorubicin resistance. 3A.** TPL2 expression levels in different human GC cell lines were determined by western blotting. **3B.** Western blot analysis of TPL2 knockdown in SCM1 and MKN45 cells. Cells were transfected with either scrambled siRNA (scr; 5 μg/mL) or TPL2-specific siRNAs (siTPL2#1 and siTPL2#2; 5 μg/mL each), as well as with a non-targeting shRNA control (shNC; 5 μg/mL) or TPL2-specific shRNAs (shTPL2#1 and shTPL2#2; 5 μg/mL each). After 24 hours, cells were harvested and protein lysates were prepared for analysis of TPL2 expression. GAPDH was used as an internal loading control. TPL2 protein was detected using a specific primary antibody. The results demonstrate effective knockdown of TPL2 in gastric cancer cells, supporting the specificity of the observed protein signal. **3C.** MTS analysis of TPL2 knockdown in SCM1 and MKN45 cells (n = 6). *p < 0.05. **3D.** Thymidine incorporation was used to observe cell proliferation after TPL2. knockdown in SCM1 or MKN45 cells. The number of counting cells was recorded (n = 6). *p < 0.05. **3E.** Representative images of tumors removed from nude mice. All mice were initially inoculated with the same number of cancer cells (1 × 10⁶) injected into the abdominal cavity. Tumors were allowed to establish for 7 days following intraperitoneal injection of either MKN45 or SCM1 cells. Starting on day 7, mice were treated intraperitoneally (i.p.) with a TPL2 inhibitor (5 mg/kg, twice per week) for 30 days. In a separate experiment, mice were implanted with either MKN45/shTPL2 cells (n = 5) or control MKN45 cells (n = 5) and monitored for 4 weeks. Tumors were then harvested for imaging and further analysis. “I” and “II” denote the subgroups as follows: **a.** Inoculation with gastric cancer SCM1 cells transfected with either group I: shEGF or group II: shTPL2; **b.** Inoculation with gastric cancer MKN45 cells transfected with either group I: shEGF or group II: shTPL2; **c.** Inoculation with gastric cancer SCM1 cells treated with either group I: control or group II: TPL2 inhibitor (inh); **d.** Inoculation with gastric cancer MKN45 cells treated with either group I: control or group II: TPL2 inhibitor (inh). Subgroups a, b, c, and d in Figure 3E were quantified and presented in Figure 3F. **3F.** Quantification of tumor weight and tumor volume from Figure 3E were analyzed compared with the respective control groups. The results shown are representative at least five mice numbers per group. All experiments were repeated at least three times. *p < 0.05. **3G.** Colony formation assay of TPL2 knockdown or inhibitor in SCM1 cells with or without chemotherapy *doxorubicin* treatment (100 nM). “I” and “II” denote the subgroups as follows: **a.** Inoculation with gastric cancer SCM1 cells transfected with either group I: shEGF or group II: shTPL2; **b.** Inoculation with gastric cancer SCM1 cells treated with either group I: control or group II: TPL2 inhibitor (inh); **c.** Inoculation with gastric cancer SCM1 cells transfected with either Group I: shEGF or Group II: shTPL2, followed by doxorubicin treatment (100 nM). **d.** Inoculation with gastric cancer SCM1 cells treated with either Group I: control or Group II: TPL2 inhibitor (inh), followed by doxorubicin treatment (100 nM). Subgroups a, b, c, and d in Figure 3G were quantified and presented in Figure 3H. **3H.** Quantification in colony formation assay from Figure 3G was conducted for TPL2 knockdown in SCM1 and MKN45 cells, with or without doxorubicin treatment (100 nM) (n = 6). Statistical significance is indicated as *p < 0.05.

As demonstrated by western blotting in Fig. 3A, TPL2 expression varied in GC cell lines, both SCM1 and MKN45 showed high protein expression, while AGS, N87, SNU5, SNU16, KATOIII, TMK-1 had relatively low TPL2 expression. To explore the biological function of TPL2 in GC, a transient transfection model using both siRNAs and shRNAs using Lipofectin transfection in SCM1 and MKN45 cells were established, which successfully downregulated the expression of TPL2 (Fig. 3B; Supplementary Fig. 1). Then, the behaviors of SCM1 and MKN45 cells were examined by MTS and thymidine incorporation assay. The downregulation of TPL2 significantly restrained the cell viability of both SCM1 and MKN45 cells compared to control groups in a 5-day period (Fig. 3C). Moreover, a similar pattern was observed in cells with lower TPL2 expression levels, such as N87, SUN5, and KATO3 (Supplementary Fig. 2). In parallel, the ratio of thymidine incorporation counting rate was decreased in the siTPL2, shTPL2 or TPL2 inhibitor in SCM1or MKN45 group compared with the control group (Fig. 3D). Simultaneously, specific TPL2 pharmacological inhibitor were shown the similar pattern (Supplementary Fig. 3). Our results showed that both TPL2 knockdown and pharmacological inhibition reduced cell viability compared to controls, indicating that TPL2 plays a role in supporting both proliferation and survival of these cells. The essential role of TPL2 for cell proliferation was further confirmed in subcutaneous xenograft tumor models. As shown in Fig. 3E, the tumorigenicity of shTPL2-SCM1 group was markedly reduced since lower tumor weight and smaller tumor volume were observed after 4 weeks under silencing of TPL2 (a-b) or exposure to TPL2 inhibitor (c-d). In Figure 3F, tumor weight and volume from Figure 3E were quantified and compared with the respective control groups. In addition, shTPL2 (a-b) or exposure to TPL2 inhibitor exhibited a sensitize to doxorubicin treatment as demonstrated in the colony formation assays in SCM1 cells (Fig. 3G). In Figure 3H, quantification of the colony formation assay from Figure 3G was conducted for TPL2 knockdown in SCM1 and MKN45 cells, with or without doxorubicin treatment (100 nM) (n = 6). Statistical significance is indicated as *p < 0.05. Moreover, MKN45 cells, which are derived from signet-ring cell carcinoma and are particularly sensitive to chemotherapeutic agents such as doxorubicin and 5-FU, have been included in the Supplementary Fig. 18. These findings revealed that TPL2 silencing or TPL2 inhibitor dampens proliferation and chemotherapy doxorubicin treatment resistance of GC cells.

### TPL2 overexpression promotes GC cell proliferation and chemotherapy resistance

**Figure 4:**
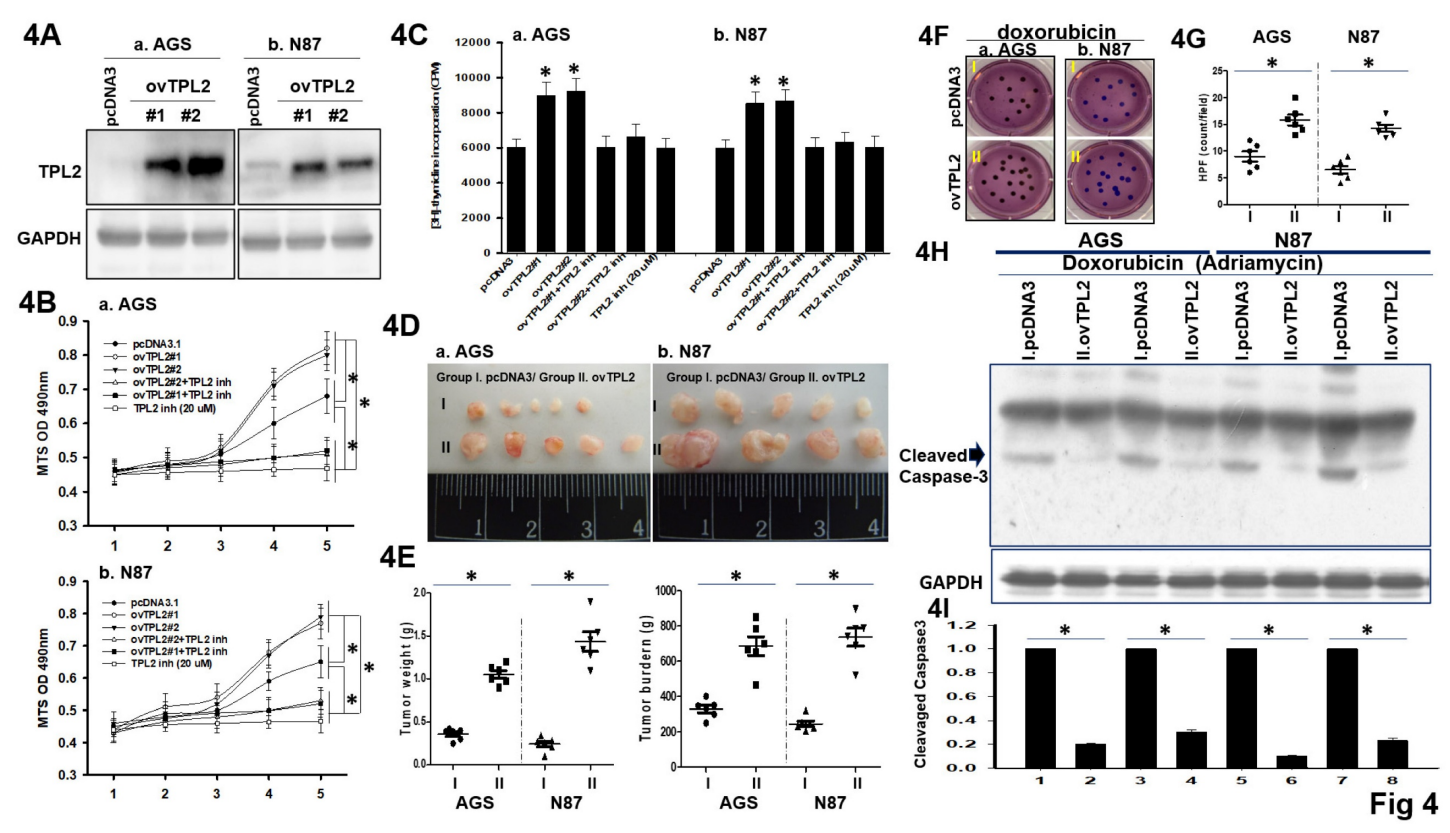
** The effects of TPL2 upregulation on GC cell proliferation, tumorigenicity and Doxorubicin resistance. 4A.** Western blot results of TPL2 overexpression in AGS and N87 cells. Anti- TPL2 (left) and anti- TPL2 (right) antibodies were used. **4B.** The growth rates of AGS and N87 cells with or without TPL2 overexpression and TPL2 inhibitor were monitored by MTS assay (n = 6). *p < 0.05. **4C.** Thymidine incorporation assay was used to detect cell proliferation in AGS and N87 cells with TPL2 overexpression, TPL2 inhibitor and control cells. The number of quantification cells was recorded (n = 6). *p < 0.05. **4D.** Representative images of tumors excised from nude mice implanted with AGS or N87 cells, either with TPL2 overexpression (ovTPL2; n = 6) or control vector (pcDNA3; n = 8) after 3 weeks. All mice were initially inoculated with an equal number of cancer cells (1 × 10⁶) via intraperitoneal injection. Tumor weight and volume were analyzed and compared to the control group. "I" and "II" indicate the following subgroups: Group I: pcDNA3 (control), Group II: ovTPL2 (TPL2 overexpression). **4E.** Quantification of tumor weight (left) and tumor burden (right) (n = 6). *p < 0.05. **4F.** Under doxorubicin treatment (50 nM), colony formation assays were performed using AGS and N87 gastric cancer cells with or without TPL2 overexpression (ovTPL2; 5 μg/ml). Cells were transfected with either the control vector pcDNA3 or TPL2 overexpression plasmid and then treated with doxorubicin. Group I: pcDNA3 (control), Group II: ovTPL2 (TPL2 overexpression). **4G.** Quantification of TPL2 overexpressed AGS, N87 cells and control cells with or without Doxorubicin treatment (50 nM) (n = 6). *p < 0.05. **4H.** Western blot analysis of apoptotic AGS, N87 cells with or without Doxorubicin treatment (50 nM). TPL2 expression cells had an anti-apoptosis effect. GAPDH take as the internal control. **4I.** Quantification of Cleavage caspase3 form expression profile (n = 6). *p < 0.05.

To examine the propose made above, two overexpressed TPL2 GC cell lines (AGS/ovTPL2-#1 and AGS/ovTPL2-#2, N87/ovTPL2-#1, N87/ovTPL2-#2) established by several options are used for the generation, depending on the serial section and scope of the experiment method (Fig. 4A). Simultaneously, MTS assays and [³H]-thymidine incorporation assays were performed with and without TPL2 inhibitors. The results showed a significant decrease in apoptosis and an increase in cell proliferation (Fig. 4B-4C), suggesting that TPL2 overexpression promotes the proliferation of AGS and N87 cells and that this effect depends on TPL2 activity. Moreover, the role of TPL2 in regulating the MEK pathway has also been examined and is presented in Supplementary Fig. 4, where TPL2 was found to be involved in the activation of this signaling cascade. Consistently, thymidine incorporation assay and *in vivo* subcutaneous tumor xenograft assay also confirmed that TPL2 overexpression promoted GC cell proliferation and played a crucial role in tumor growth (Fig. 4C, 4D). Quantification of tumor weight (left) and tumor burden (right) shown in Fig. 4E. To test whether TPL2 overexpression influences Doxorubicin (Adriamycin) resistance, AGS/ovTPL2 cells were then treated with Doxorubicin followed by colony formation assay. The results showed that overexpression of TPL2 enhanced the resistance of AGS, N87 cells to Doxorubicin (Fig. 4F). Quantification analysis presented in Fig. 4G. Western blotting analysis was used to investigate whether TPL2 overexpression affects Doxorubicin-induced Cleaved Caspase-3 result in apoptosis. Compared with the control group, AGS/ovTPL2 or N871/ovTPL2 cells showed a significantly decreased rate of apoptosis upon treatment with Doxorubicin (Fig. 4H), implicating that the Doxorubicin-induced apoptosis was weakened by overexpression of TPL2.

### TPL2 affects hypoxia signaling pathway by interacting with PPARδ

**Figure 5:**
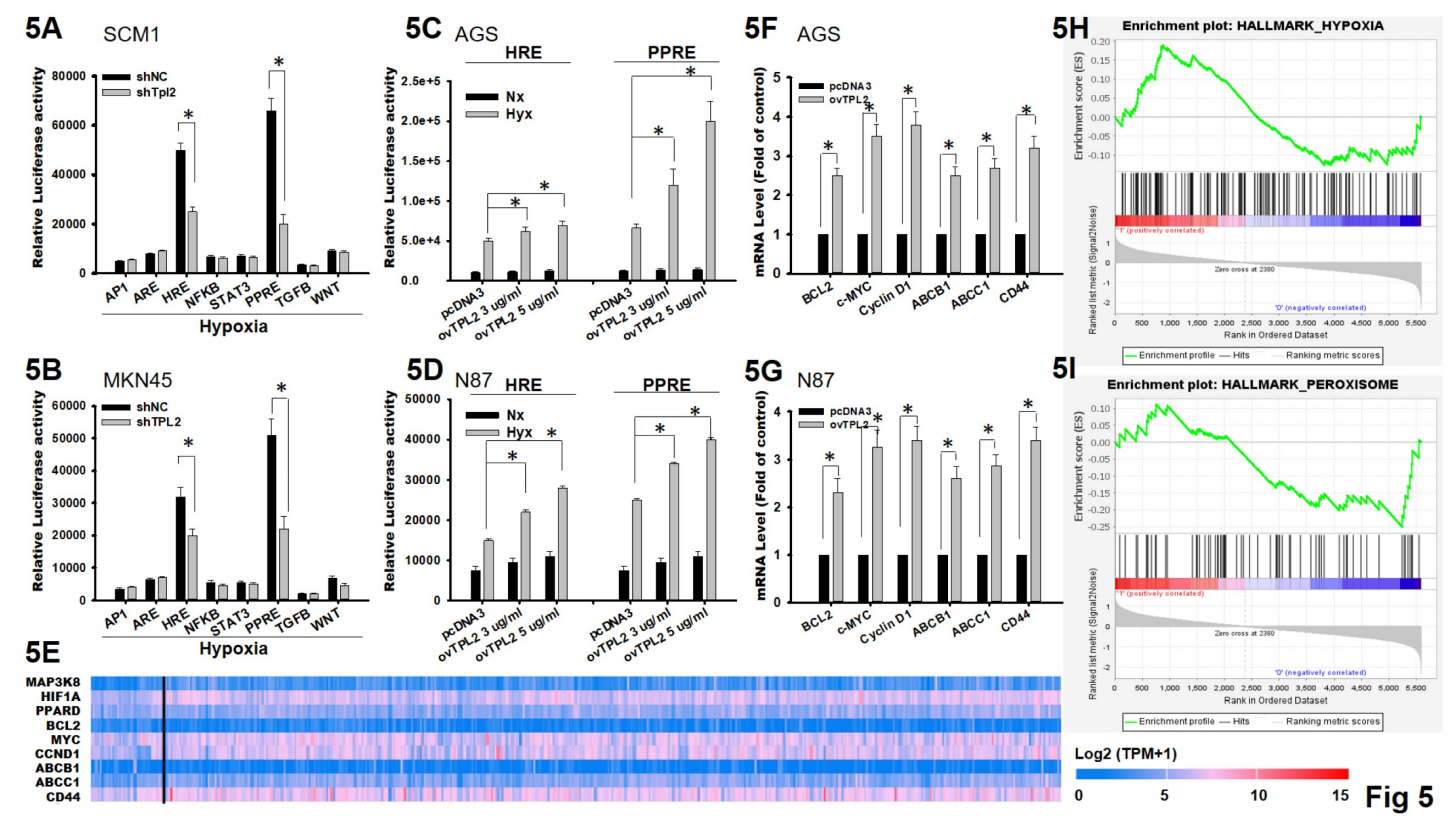
** TPL2 was involved in the regulation of HIF1α/PPARδ pathway. 5A.** Dual-luciferase reporter assays were performed in SCM1/shTPL2 cells with eight typical signaling pathway reporter systems as indicated (n = 6). *p < 0.05. **5B.** Dual-luciferase reporter assays were presented in MKN45/shTPL2 cells with eight typical signaling pathway reporter systems as indicated (n = 6). *p < 0.05. **5C.** Dual-luciferase reporter assay for HIF1α/PPARδ pathway in AGS/ovTPL2 cells after hypoxia treatment (n = 6). *p < 0.05. **5D.** Dual-luciferase reporter assay for HIF1α/PPARδ axis in N87/ovTPL2 cells after hypoxia exposure (n = 6). *p < 0.05. **5E.** Expression pattern of input genes (MAP3K8, HIF1A, PPARD, BCL2, MYC, CCND1, ABCB1, ABCC1, CD44) in gastric cancer adenocarcinoma. **5F.** Relative mRNA expression of BCL2, c-MYC, Cyclin D1, ABCB1, ABCC1 and CD44 in AGS/ovTPL2 cells by RT-qPCR (n = 6). *p < 0.05. **5G.** Relative mRNA expression of BCL2, c-MYC, Cyclin D1, ABCB1, ABCC1 and CD44 in N87/ovTPL2 cells by RT-qPCR (n = 6). *p < 0.05. **5H.** GSEA analysis comparing the gene sets of hypoxia pathway with TPL2 expression. Data were obtained from TCGA database. **I.** GSEA analysis (GSE2865) comparing the gene sets of peroxisome pathway with TPL2 expression. Data were obtained from TCGA database.

Cancer resistance is a complexity phenomenon involving verity mechanisms, and hypoxia is one of the main features of solid tumors that affect the cellular expression program and enhances chemoresistance. To explore the mechanism by which TPL2 promotes tumor growth and drug resistance, dual-luciferase reporter assays were performed under hypoxia condition. Among eight signaling pathways, only HRE and PPRE pathway was significantly influenced and exhibited reduced relative luciferase activity after TPL2 knockdown in SCM1 and MKN45, suggesting a possible involvement of TPL2 in the PPRE pathway, and hypoxia targets HRE and PPRE pathway and subsequently to magnify the luciferase activity (Fig. 5A-5B). In addition, overexpression of TPL2 markedly increased hypoxia-induced HRE and PPRE pathway activation (Fig. 5C-5D). We further to screen and identify the expression pattern of input genes in gastric cancer adenocarcinoma in growth and chemoresistance gene (Fig. 5E). Next, we analyzed the expression of downstream targets of proliferation gene by BCL2, c-MYC (MYC*)*, Cyclin D1 (CCND1) and chemoresistance candidate ABCB1, ABCC1 and CD44 using qRT-PCR. Compared to the control group, a significant upregulation of the related factors was found individually, when TPL2 was overexpressed AGS cells. Similar results were observed in TPL2 overexpressed N87 cells (Fig. 5F). Both pharmacological inhibition and genetic manipulation, either TPL2 overexpression or shTPL2 knockdown, produced consistent results as described above (Supplementary Fig. 5). In the meantime, Gene Set Enrichment Analysis (GSEA) was performed using TCGA-STAD datasets (GSE2865) and verified the positive correlation of TPL2 with hypoxia and peroxisome signaling (Fig. 5F).

**Figure 6:**
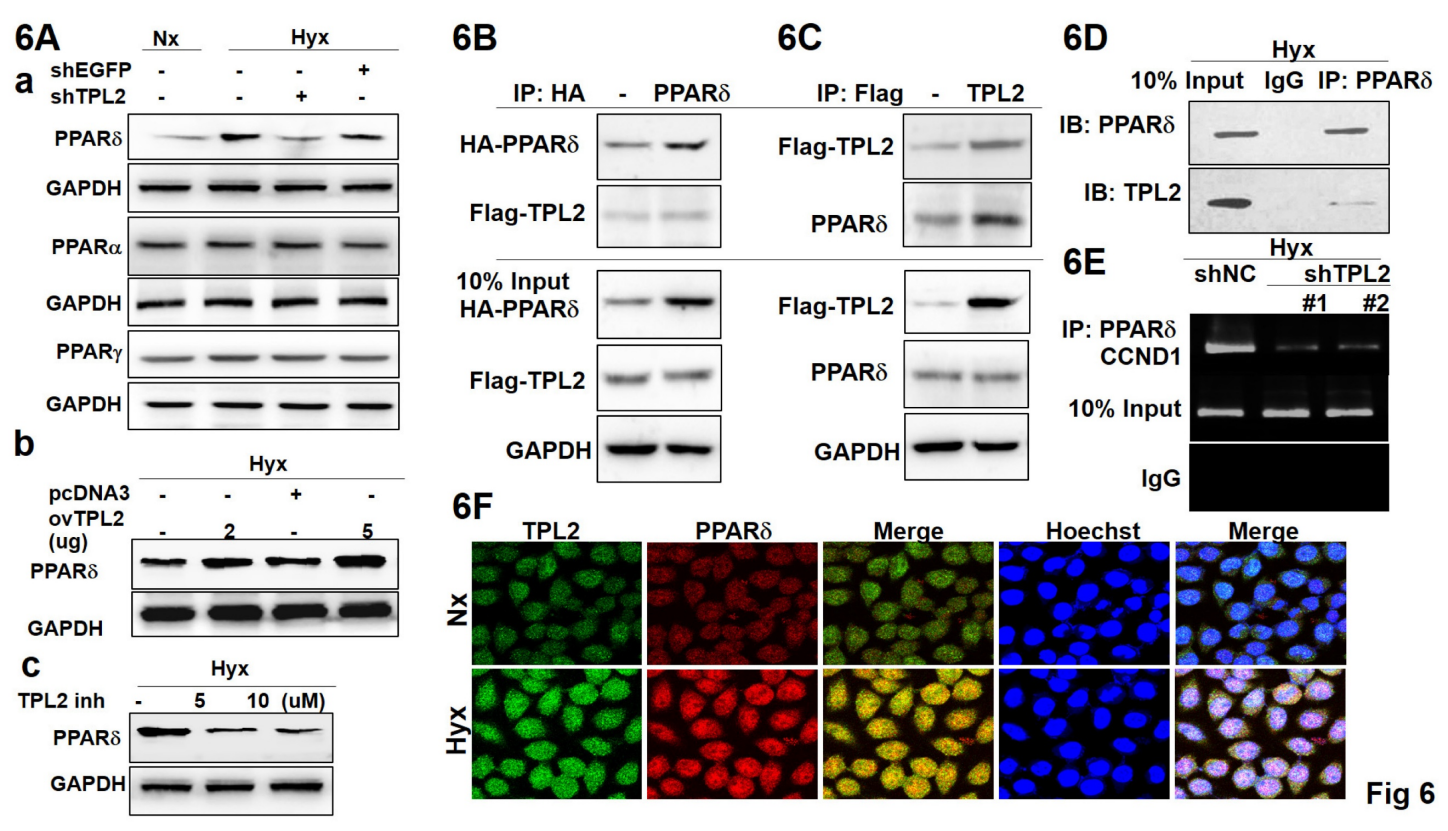
** TPL2 was participated in the targeting of HIF1α/PPARδ axis.** Gastric cancer cell transfection shEGFP, shMAP3K8 (shTPL2), pcDNA3, and pDONR223-MAP3K8 (ovTPL2) or TPL2 inhibitor overnight following hypoxia treated. **6A.** Western blot analysis of PPARα, PPARβ, and PPARδ protein expression under normoxic or hypoxic conditions as indicated. **6B.** Co-IP assays were performed in SCM1/TPL2 cells transiently transfected with HA-PPARδ plasmid. **6C.** Exogenous TPL2 was immunoprecipitated with the anti-HA antibody. **6D.** Protein-protein interaction by immunoprecipitation assay. Endogenous PPARδ was pulled down with anti-FLAG antibody in SCM1/TPL2-2 cells via Co-IP analysis. **6E.** Chromatin immunoprecipitation (ChIP) was used to evaluate protein-gene interactions. Endogenous TPL2 was coimmunoprecipitated with anti-PPARδ antibody in SCM1 cells. Results of ChIP assay conducted using chromatins isolated from SCM1 cells with TPL2 knockdown and control cells. The immunoprecipitated DNA by anti-PPARδ antibody was analyzed by agarose gel electrophoresis. Normal IgG was used as a negative control. **6F.** Co-localization of both TPL2 (red) and PPARδ (green) in SCM1/TPL2 cells was shown. Scale bar: 30 μm. All of the results shown are representative at least five independent experiments.

To gain more insights into the regulation of PPARδ signaling by TPL2, we detected the PPARδ expression when TPL2 was silenced or overexpressed. As a result, the PPARδ protein levels were significantly changed with TPL2 expression alteration under hypoxia condition (Fig. 6A-a), but not PPARα or PPARγ protein (data not shown). Overexertion of TPL2 augmented PPARδ production, and inhibition of TPL2 abated PPARδ amount (Fig. 6A-bc). Quantification analysis shown in Supplementary Fig. 6. Simultaneously, these effects were found to depend on the kinase activity of TPL2, as demonstrated using a TPL2 inhibitor in PPARδ luciferase reporter assays and Western blot analysis (Supplementary Figs. 7 and 8). Moreover, we examined whether the two proteins have an interaction by co-immunoprecipitation experiments. Exogenous FLAG-tagged TPL2 was pulled down with HA-tagged PPARδ, and likewise PPARδ was detected in FLAG-tagged TPL2 immunoprecipitated complex (Fig. 6B-6C). Remarkably, TPL2 was also seen in endogenous immunoprecipitated PPARδ complex (Fig. 6D). More importantly, ChIP assays showed that TPL2 downregulation decreased the binding of PPARδ to the Cyclin D1 promoter region that was proved by previous reports as PPARδ binding site (Fig. 6E). According to the above-mentioned reported, confocal assays further demonstrated that the two proteins had a co-localization in SCM1 cells (Fig. 6F). Staining specific and knockdown efficiency was also confirmed by immunofluorescence staining, as shown in Supplementary Fig. 9. These data suggest that TPL2 and PPARδ interact with each other, and the evidence for their association is derived from a combination of complementary approaches—not solely from confocal co-localization. In particular, co-immunoprecipitation assays (Figures 6B-D; Supplementary Fig. 19) and functional analyses (Supplementary Figs. 7 and 8) provide biochemical and mechanistic support for a specific and functionally relevant interaction between the two proteins. Taken together, these findings implicated that TPL2 is involved in the regulation of PPARδ signaling and affects PPARδ activity by interaction with PPARδ.

### PPARδ significantly regulates TPL2 expression in GC cells

**Figure 7:**
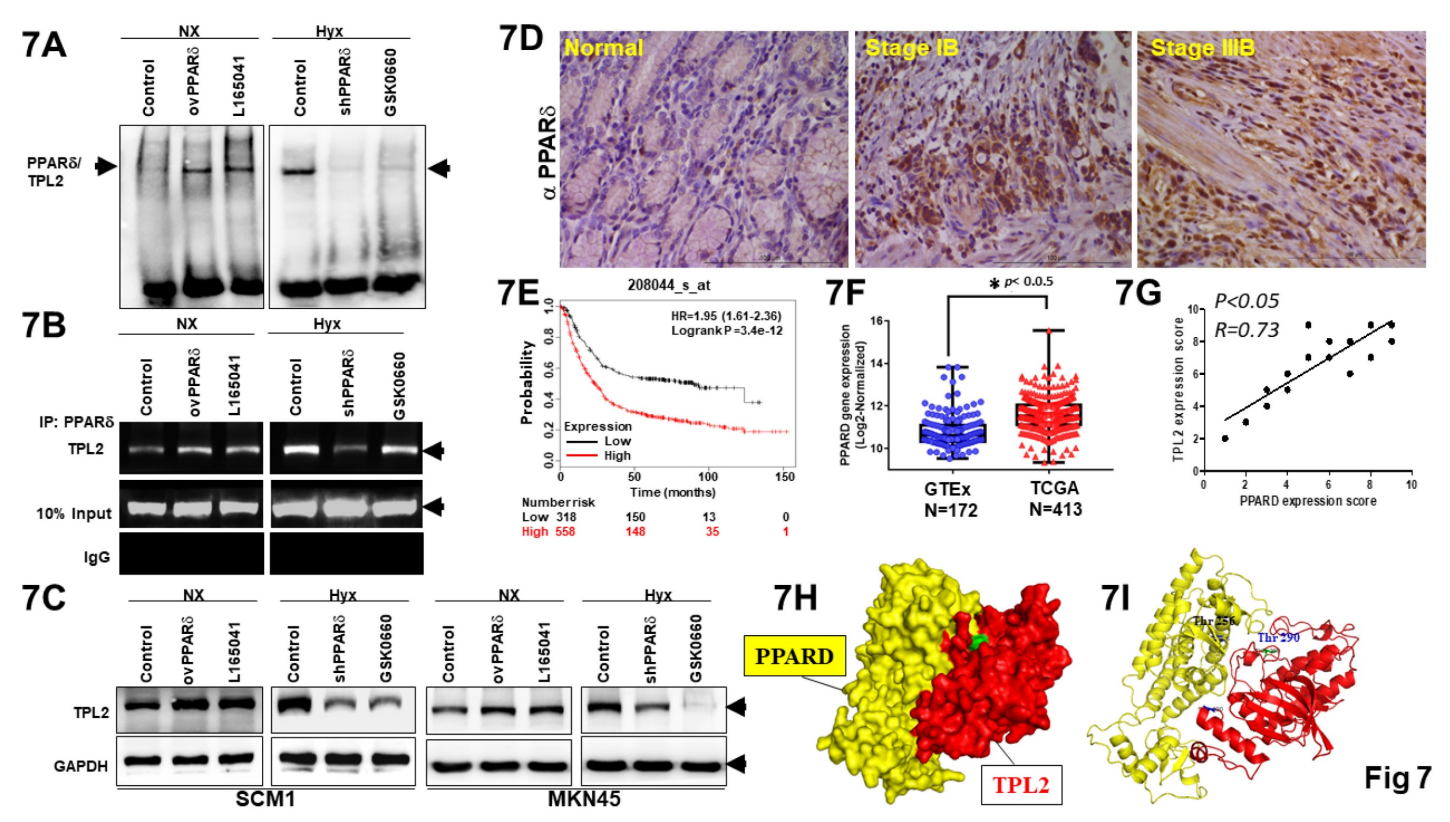
** TPL2 was significantly regulated by PPARδ.** Overexpression or shRNA PPARδ plasmids were transfected into SCM1 cells. Meanwhile, L-165,041, potent PPARδ agonist and GSK 0660, selective PPARδ antagonist also exposure to evaluate effect. **7A.** Two putative consensus-binding sites of PPARδ were shown in the TPL2 promoter region (-1940 to -1920 bp, -385 to -365 bp). Then the DNA binding activity was examined by EMSA assay. The results shown are representative at least five independent experiments. **7B.** CHIP assay of the TPL2 promoter reporters in SCM1 cells when overexpression or shRNA PPARδ was transfected. The results shown are representative at least five independent experiments. **7C.** Western blot detection for TPL2 expression. The relative protein levels of TPL2 in MKN45 and SCM1 cells transfected with ovPPARδ or shPPARδ plasmid and pharmacological induction by L-165,041 and GSK 0660. The results shown are representative at least five independent experiments. **7D.** PPARδ is upregulated in gastric cancer tissues from the Taichung Veterans General Hospital (TCVGH) Tissues Bank (*n* = 45). (a) Representative immuno-staining of PPARδ expressions in human normal gastric mucosa. (b) Poorly differentiated intestinal adenocarcinoma, (c) diffused adenocarcinoma in mucosa. Scale bar = 100 μm. **7E.** High PPARδ expressions were associated with decreased overall survival probability in gastric cancer. Data obtained were from the dataset (208044_s_at, *n* = 875) through a comprehensive search using Kaplan-Meier plotter.com for PPARδ evaluation. The explores and presents data from the public web server KM Plotter for gastric cancer (https://kmplot.com/analysis/index.php?p=service&cancer=gastric). **7F.** PPARδ expression levels in normal tissues and tumor samples derived from publicly available Genotype-Tissue Expression (GTEx, *n* = 172) and The Cancer Genome Atlas (TCGA, *n* = 413) gene expression data, respectively, plotted as box and whisker plots. OS stratified by quartiles distribution. *p < 0.05. **7G.** Assessment of the correlation between TPL2 and PPARδ expression in GC specimens (n = 30) using Pearson correlation coefficient analysis. Some of the dots on the graph represent more than one specimen. **7H.** Surface representation of the protein-protein interaction. TPL2 (red) and PPARδ (yellow) are shown in surface representation. **7I.** Local interaction positions of the TPL2 (Thr 290) and PPARδ (Thr 256) active site.

Interestingly, two putative consensus binding sites of PPARδ (5'-AGGTCA-3') were found on TPL2 promoter region located at -1930 and -375bp upstream of the transcriptional starting site. To assess whether PPARδ regulates TPL2 expression, we transfection shPPARδ, ovPPARδ plasmid and potent pharmacological PPARδ agonist, L-165,041, as well as GSK 0660, selective PPARδ antagonist to evaluate DNA binding activity by electrophoretic mobility shift assay (EMSA) and DNA-protein interactions by Chromatin immunoprecipitation (ChIP) assays, respectively. As a result of EMSA for PPARδ binding site significantly attenuated TPL2 promoter site activity in SCM1 cells, especially under shPPARδ and GSK0660 were exposure. The concordant reciprocal results could also be seen that PPARδ overexpression or L-165,041 had promoted effect on TPL2 DNA binding activity (Fig. 7A). To provide more direct evidence, we conducted a ChIP assay using chromatins prepared from SCM1 cells. The results indicated that ovPPARδ or endogenous PPARδ bound to the putative TPL2 binding sites (Fig. 7B). To confirm that PPARd regulates TPL2 transcription directly, reporter assays also be carried out. Mutation of the PPRE element significantly attenuated PPARδ-induced promoter activation in both AGS and SCM1 cells (Supplementary Fig. 10). Moreover, western blot results showed that TPL2 expression levels were changed with PPARδ knockdown or overexpression. Meanwhile, L-165,041, potent PPARδ agonist markedly increased TPL2 expression in SCM1 and MKN45 cells. GSK 0660, selective PPARδ antagonist reduced TPL2 expression. These data suggested that TPL2 is regulated by PPARδ at transcriptional level. (Fig. 7C) Quantification analysis shown in Supplementary Fig. 11. In addition, a positive association between PPARδ and TPL2 expression was observed in a clinical subjects of tissue section by immunostaining with PPARδ antibody (Fig. 7D). In addition, by web-based correlation, there was a markedly positive correlation between the low expression levels of PPARδ in 318; high expression levels of PPARδ in 558 patients with gastric cancer, using the selected parameters and run on by Kaplan-Meier plotter (KMplot.com), Probability GSE208044 dataset, or by TCGA (Fig. 7E). Simultaneously, data from the Genotype-Tissue Expression project and the Cancer Genome Atlas were first integrated to comprehensively analyze the transcriptomes of 172 healthy and 413 tumor tissues (Fig. 7F). Importantly, a positive association between PPARδ and TPL2 expression was observed in a 30 cases of tissue microarray by immunostaining with TPL2 antibody and PPARδ antibody (Fig. 7G). Surface representation of the protein-protein interaction and local interaction positions of the TPL2 (Thr 290) and PPARδ (Thr 256) active site by structure biology analysis was shown in Fig. 7H-7I. DSC serves as both a sensitive detector of protein-protein binding events and a quantitative method to probe the energetics of such interactions. In our experiments, we observed a rightward shift in the thermal transition peak, indicating increased TPL2/PPARD protein stability, providing to binding or complex formation (Supplementary Fig. 12). To confirm the physical interaction, we performed an in vitro GST pulldown assay. As shown in Supplementary Fig. 20, GST-TPL2[30-397] successfully pulled down PPARδ, while GST alone did not. Additionally, no signal was detected in control reactions lacking PPARδ, supporting the specificity of the interaction. This result validates that the truncated TPL2 protein used in DSC experiments retains the ability to bind PPARδ. Collectively, these data strongly indicated that PPARδ can significantly regulate TPL2 expression.

### PPARδ knockdown decreases TPL2-induced cell proliferation and chemo-resistance and vice versa

**Figure 8:**
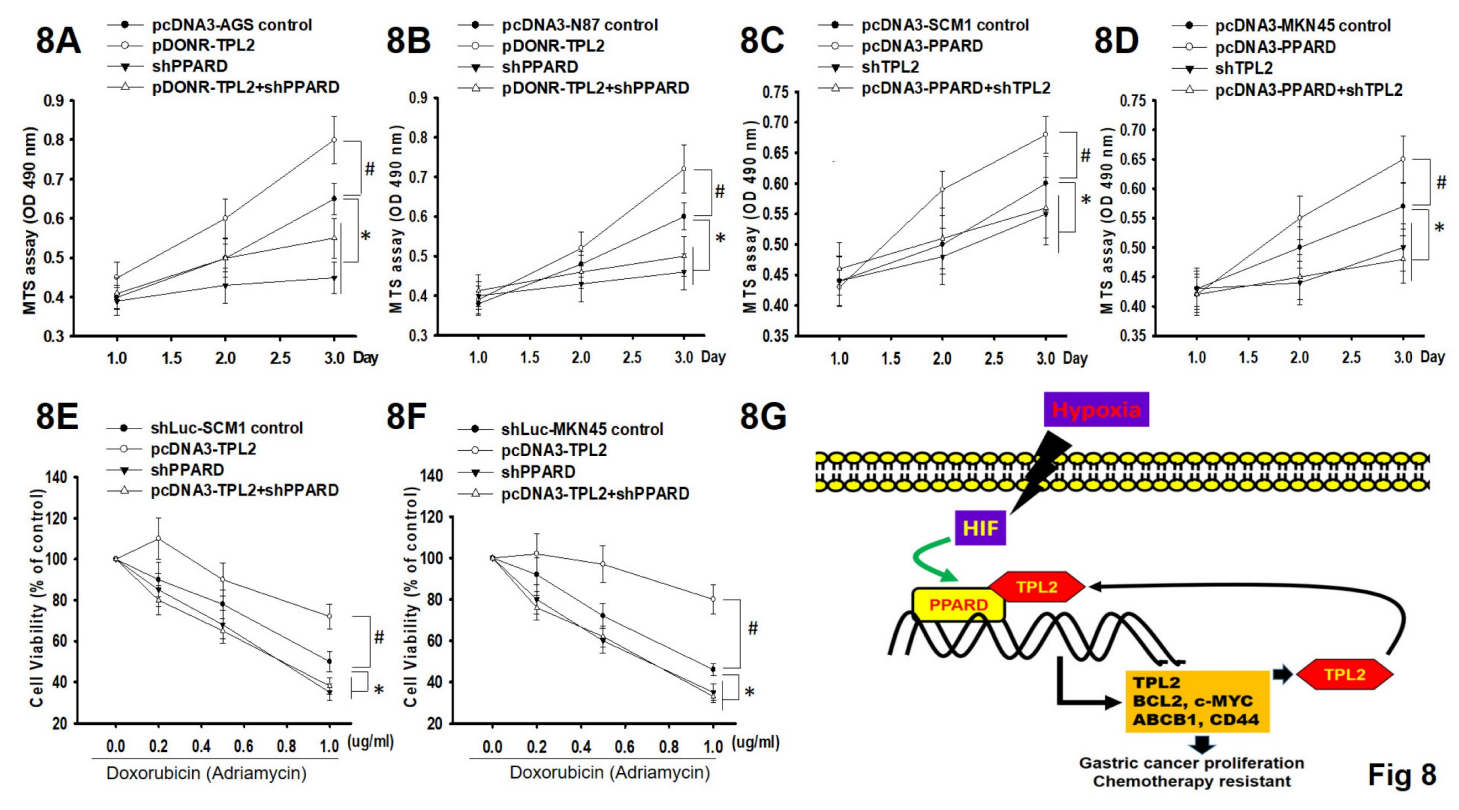
** The functional relationship between TPL2 and PPARδ in regulating cell proliferation and Doxorubicin resistance. 8A.** Cell viability was measured by MTS assay in AGS/TPL2 with or without PPARδ knockdown. #P<0.05 compared with control group. *P < 0.05 compared with control group. **8B.** Cell viability was measured by MTS assay in N87/ TPL2 cells with or without PPARδ knockdown. #P<0.05 compared with control group. *P < 0.05 compared with control group. **8C.** Under hypoxia culture conditions, MTS assay in SCM1 cells transfected with pcDNA3-PPARδ, shTPL2, empty vector or pcDNA3 plasmid in a combined way as indicated. #P<0.05 compared with control group. *P < 0.05 compared with control group. **8D.** Under hypoxia culture conditions, MTS assay in MKN45 cells transfected with pcDNA3-PPARδ, shTPL2, empty vector or pcDNA3 plasmid in a combined way as indicated. #P<0.05 compared with control group. *P < 0.05 compared with control group. **8E.** Cell viability was examined by MTS under gradient Doxorubicin treatment using SCM1 cells as indicated. #P<0.05 compared with control group. *P < 0.05 compared with control group. **8F.** Cell viability was measured by MTS under gradient Doxorubicin treatment using MKN45cells as indicated. #P<0.05 compared with control group. *P < 0.05 compared with control group. **8G.** Schematic diagram of the positive feedback loop between TPL2 and PPARδ.

To determine the functional relationship between TPL2 and PPARδ in GC cell proliferation and chemoresistance, we silenced PPARδ in TPL2 overexpressed AGS and N87 cells. The MTS assays revealed that PPARδ silencing decreased TPL2 overexpression induced cell viability under normal culture conditions or Doxorubicin treated conditions (Fig. 8A-B; 8E-F). Simultaneously, we also silenced TPL2 in PPARδ overexpressed SCM1 and MKN45 cells. The results presented that TPL2 silencing decreased PPARδ overexpression induced cell viability under hypoxia culture conditions. (Fig. 8C-8D) As well, TPL2 knockdown in PPARδ overexpressed cells also retarded PPARδ mediated cell growth and Doxorubicin resistance. These effects require TPL2 kinase activity, as demonstrated using a TPL2 inhibitor (Supplementary Fig. 13). These observations suggest that TPL2 and PPARδ interdependently regulate cell proliferation and the response to chemotherapy. Thus, together with previous findings, we propose a TPL2/PPARδ reciprocal positive feedback loop involved in the regulation of GC cell proliferation and chemoresistance, as depicted in Fig. 8G.

## Discussion

TPL2, also known as COT or MAP3K8, was previously reported as activated downstream of TNFαR, IL1R, TLR, CD40, IL17R, and some GPCRs, which is involved in regulate a cascade of inflammatory responses 7. Emerging evidence has shown TPL2 to be a key element in variety of tumors, including prostate cancer, pancreatic cancer, and bladder cancer 35-39. In the present study, we demonstrated that TPL2 was significantly upregulated in GC cancer tissues, and highly expression of TPL2 was correlated with poor overall survival, and clinicopathological characteristics of this cancer. Moreover, we proved for the first time that TPL2 downregulation impeded GC cell proliferation and tumor growth. On the contrary, increasing of TPL2 expression significantly enhanced GC cell line proliferation and *in vivo* tumor growth. Also, TPL2 expression was positively related to the Doxorubicin resistance of GC cells, implying that TPL2 could be an anti-apoptotic factor. Given the clinical and biological significance of the TPL2, the study shown that TPL2 could be a potential biomarker for GC prognosis and could have potential application in GC therapeutics in the future.

Mechanistically, Paarth B Dodhiawala et al. described TPL2 as a novel target gene of IRAK, as the essential kinase that propels both MAPK and NF-κB cascades, and TPL2 inhibitor synergized with chemotherapy to curb PDAC growth in vivo 40. Maria Vougioukalaki et al. reported that TPL2-MEK-ERK branch by adhesion-related molecules that may have important ramifications for cancer therapy 41. TPL2 also contributes to cell metabolism reprogramming by regulating the C/EBPB, NFκB, AP-1/Snail axis, VEGF and significantly promotes gastric tumor growth and peritoneal dissemination 23. A recent study showed that TPL2 mediates oncogenic JNK signaling by LMP1 and cell survival of EBV-transformed cells, TPL2 should be considered as an attractive target for new drugs or the repurposing of existing inhibitors against EBV-induced malignancies such as post-transplant lymphoproliferative disease 42. Moreover, TPL2 knockout mice ablation suppressed hepatocellular carcinoma development by inhibiting hepatic inflammation and steatosis, which suggested that pro-inflammatory effect of Tpl2 could be a molecular target for HCC prevention and inhibiting HCC development 43. In addition, TPL2-dependent oxidative burst drives phosphorylation of extracellular signal-regulated kinase during TLR3 and TLR 9 signaling. It could be further confirming the importance of TPL2 in innate host defense mechanisms and elucidate immune system tailors pathogen-specific gene expression patterns 44. In the present study, we found a new signaling pathway that TPL2 may be involved during cancer cell proliferation and chemoresistance through intensifying hypoxia condition. According to dual-luciferase reporter assay screening, we found that TPL2 expression was highly related to PPARδ and HRE signaling. Downregulation of TPL2 markedly dampened the activity of this signaling, implicating the involvement of TPL2 in the pathway. On the other hand, we also provided evidence that TPL2 was regulated by PPARδ. By promoter sequence analysis, we observed one putative binding sites of PPARδ in the TPL2 promoter; ChIP assays confirmed the binding of PPARδ to the putative promoter region. qRT-PCR and western blot data supported the speculation that PPARδ can transcriptionally increase TPL2 expression. *Hence*, we are the first to propose a new TPL2/ PPARδ reciprocal positive feedback loop to augment PPARδ signaling and lead to GC development and progression (Supplementary Fig. 14).

Doxorubicin is one of the important agents against gastric cancer, which the mechanisms of anticancer pharmacodynamics are via (i) intercalation into DNA and disruption of topoisomerase-II-mediated DNA repair and (ii) generation of free radicals and their damage to cellular membranes, DNA, and proteins (Thorn et al., 2011). Acquired Doxorubicin drug-resistance severely impedes the chemotherapeutic effect through modulating PTEN/Akt signaling pathway or related multiple signaling pathways, invariably leading to poor prognosis (Xu et al., 2017; Xu et al., 2018). Importantly, Doxorubicin induced HIF1a expression under normoxic condition, and it was exacerbated by hypoxia (Supplementary Fig. 15). These results were consistent with the previous reports in tumor cells upregulating normoxic HIF-1a in response to Doxorubicin (Cao et al., 2013; Osada-Oka et al., 2022). In addition, abovementioned effect was consistent with PPARδ and TPL2 kinase production. In animal validation effects, we demonstrated that knockdown TPL2 enhanced Doxorubicin medication effect; overexpression of TPL2 resisted Doxorubicin therapy effects (Supplementary Fig. 16). These results emphasize the hypothesis that the TPL2/PPARδ-targeting strategy may block chemoresistance efficacy and tumor progression.

The TPL2 gene indeed encodes two isoforms: the full-length M1-TPL2 (p58) and the shorter M30-TPL2 (p52), both translated from the same mRNA via alternative translational initiation at methionine 1 (M1) and methionine 30 (M30), respectively. Typically, when both isoforms are present and the antibody used is capable of recognizing them, two distinct bands can be observed by western blotting. In our previous studies, we have identified the key reasons that may explain the presence of a single band in Figures 3A and 3B: A in isoform stability. The M1 isoform (p58) is often unstable and subject to rapid proteasomal degradation, especially under specific cellular conditions. In earlier experiments, we treated samples with the proteasome inhibitor MG132 and observed the reappearance of the second band, supporting the notion that the M1 isoform is degraded in untreated conditions. In our experiments (Figures 3A and 3B), this is likely due to the predominant expression and/or higher stability of the M30 isoform in gastric cancer cells under the experimental conditions used. It is also possible that the longer isoform is expressed at lower levels or is more rapidly degraded, and thus below the detection limit of our assay.

In the context of tumors, the interaction between TPL2 (Tumor Progression Locus 2) and NF-κB1 p105 plays a complex and potentially dual role in regulating inflammation, cell survival, and tumor progression 45-48. TPL2 is a MAP3K that activates the MEK/ERK signaling pathway, promoting the expression of pro-inflammatory and pro-survival genes. Under normal conditions, NF-κB1 p105 binds TPL2 in a stoichiometric, high-affinity complex, inhibiting its kinase activity and preventing uncontrolled signaling. In tumors, dysregulation of this interaction—either through enhanced phosphorylation and degradation of p105 or overexpression of TPL2—can lead to constitutive activation of ERK signaling, contributing to tumor cell proliferation, resistance to apoptosis, and a pro-tumorigenic inflammatory microenvironment. Moreover, aberrant NF-κB signaling through p105 processing can further drive chronic inflammation and support oncogenic processes. In the Supplementary Fig. 17, we found that in hypoxia condition, NF-κB1 p105 is phosphorylated at specific serine residues (notably Ser337 in human p105), inhibition of TPL2 blocked this effect. In addition, MEK inhibitor could attenuated cell proliferation (Supplementary Fig. 4 and Supplementary Fig. 5) and related signaling cascade (Supplementary Fig. 7). Thus, the TPL2-p105 axis represents a critical regulatory node linking inflammation and cancer, with its disruption potentially promoting tumor development and progression.

A considerable amount of literature has demonstrated that pleiotropic effects of PPARδ is extensively involved in tumorigenesis, progression, and invasion 49-51. Yi Liu et al demonstrated that PPARδ pathway potentiated β-catenin activation in intestinal epithelial cells (IECs) via upregulation of BMP7/TAK1 signaling and promoted tumor progression and invasion by also upregulating multiple other vital pro-tumorigenic proteins, including PDGFRβ, AKT1, EIF4G1, and CDK1 in CRC 49. Moreover, PPARδ activation induces KRAS^mu^ pancreatic epithelial cells to secrete CCL2, which recruits immunosuppressive macrophages and myeloid-derived suppressor cells into pancreas via the CCL2/CCR2 axis to orchestrate an immunosuppressive tumor microenvironment and subsequently drive PanIN progression to PDAC 50. In additional, Xiangsheng Zuo and his colleague indicated that PPARδ promotes EMT, angiogenesis, migration, invasion in lung metastases of B16-F10 melanoma cells in immunocompetent mice 51. In the light of this, our results revealed that except for gene regulation in early stage; moreover, TPL2 may reinforce the binding of PPARδ to downstream targets via their protein interaction under TPL2 (Thr290/Ser400) site and PPARδ (Thr256) site. However, the exact mechanisms of how TPL2 interacts with PPARδ are still unknown. Interestingly, *CDKN1C* (also known as *p57^kip2^*) as a PPARδ target gene and a mediator of the PPARδ-mediated inhibition of cell proliferation, which provides a possible mechanistic explanation for the observed tumor endothelial hyperplasia and deregulation of tumor angiogenesis in PPARδ (-/-) mice. That point to an unexpected essential role for PPARδ in constraining tumor endothelial cell proliferation to allow for the formation of functional tumor microvessels 52. It's worth noting that, PPARδ conferred the ability to grow in exhausted tissue culture media and survive in low-glucose and other endoplasmic reticulum stress conditions such as hypoxia, suggesting that PPARδ promotes survival of breast cancer cells in harsh metabolic conditions 53. It has been reported in the literature from Bokai Zhu et al. that PPARδ promotes oncogene-induced cellular senescence through repression of endoplasmic reticulum stress 54. Yalan Wu et al. presented hypoxia-induced PPARδ, which reciprocally enhances HIF1α stability and its downstream target genes participating in the vascular repair and restoration of vascular integrity. The interaction and regulation of PPARδ-HIF1α is critical for perfusion recovery in hindlimb ischemia 55. These mechanism plays a significant role in hypoxia associated with tumor growth and aggravated.

## Conclusion

This study demonstrates that TPL2 is overexpressed in gastric cancer (GC), as well as in other cancer types. Its upregulation is strongly associated with poor overall survival in GC patients. TPL2 promotes cell proliferation and enhances Doxorubicin resistance by amplifying hypoxia/PPARδ axis signaling. Figure 8G illustrates the signaling pathways involving hypoxia-induced PPARδ/TPL2 expression in human gastric cancer cells. Our findings provide new insights into the regulation of PPARδ signaling in GC development. Furthermore, TPL2 may serve as a potential therapeutic target, extending beyond cancer to include the treatment of diseases associated with pathological hypoxia.

## Supplementary Material

Supplementary methods, figures and tables.

## Abbreviations

TPL2: tumor progression locus 2
GC: gastric cancer
PPARδ: peroxisome proliferator-activated receptor delta
IHC: immunohistochemical
EMSA: electrophoretic mobility shift assay
ChIP: chromatin immunoprecipitation
IP: immunoprecipitation
MTS: 3-(4,5-dimethylthiazol-2-yl)-5-(3-carboxymethoxyphenyl)-2-(4-sulfophenyl)-2H-tetrazolium, inner salt
qRT-PCR: quantitative real-time PCR
TPM: transcripts per million
PPRE: peroxisome proliferator response element
OS: overall patient survival
FP: first progression
PPS: post progression survival
CCND1: cyclin D1
GSEA: gene set enrichment analysis
