# Supplementary material for: TPL2 Promotes Gastric Cancer Progression and Chemoresistance Through a Hypoxia-Induced Positive Feedback Loop with PPARδ
Source: Int J Biol Sci. 2025 Sep 12;21(13):5874–90. doi: 10.7150/ijbs.111998 (PMC12509916; doi:10.7150/ijbs.111998)
Supplement: Supplementary file 1 — Supplementary methods, figures and tables. [file ijbsv21p5874s1.pdf]

**Supplementary Table 1.** Primary antibodies used in the study				
**Name**/RRID number	Sp. (Clone number or code number)	In the work usage	Vendor	Note
**TPL2**/AB_2140669	SC-720 (M-20)	WB, IP, IF, IHC	Santa Cruz Biotechnology	Cot Antibody
**Tpl2 (E1R5H)**/ AB_612839	#71184S	WB, IP	Cell Signaling	
**p-TPL2 (Thr290)**/ AB_2816364	PA5-104891	WB, IHC (P) ICC/IF	Thermo Fisher Scientific	
**p-TPL2 (Ser400)**/ AB_2817566	PA5-106168	WB, ICC/IF	Thermo Fisher Scientific	
**GAPDH**/AB_627679	SC-32233	WB	Cell Signaling	
**Caspase3**/AB_331439	#9662S	WB, IP,IHC	Cell Signaling	
**Caspase3**/ AB_630986	SC-1226	WB, IP, IF, IHC	Santa Cruz Biotechnology	
**PPARD**/AB_2268420	SC-7197 (H74)	WB, IP, IF, IHC	Santa Cruz Biotechnology	
**PPARD**/ AB_2165902	ab23673	WB, IP, IF, IHC	Abcam	
**p-PPARD (Thr256)**/AB_2844410	AF4331	WB	Affinity Biosciences	
**p-PPARD (Thr256)**/AB_2816555	PA5-105082	WB	Invitrogen	

**Hoechst 33342**/AB_10626776	62249	IF	Thermo Fisher Scientific	
Application Key: **WB**-Western Blot **IP-**Immunoprecipitation **IHC**-Immunohistochemistry **ChIP**-Chromatin Immunoprecipitation **IF**-Immunofluorescence **F**-Flow Cytometry				

**Supplementary Table 2.** Secondary antibodies used in the study				
**Name**/RRID number	Sp. (Clone number or code number)	In the work usage	Vendor	Note
Fluorescein-Labeled Antibody To Rabbit IgG (H + L)	02-15-16	IF	**K**irkegaard & **P**erry **L**aboratories, Inc	910 Clopper Road, Gaithersburg, MD 20878, USA
Fluorescein-Labeled Antibody To Mouse IgG (H + L)	172–1806	IF	Kirkegaard & Perry Laboratories, Inc	
Rhodamine (TRITC)–conjugated Goat Anti-Rabbit IgG (H+L)	03-15-06	IF	Kirkegaard & Perry Laboratories, Inc	
Rhodamine (TRITC)–conjugated Goat Anti-Mouse IgG (H+L)	03-18-06	IF	Kirkegaard & Perry Laboratories, Inc	

Peroxidase-conjugated affinipure goat anti-mouse IgG (H + L)	115-035-003	WB	Jackson ImmunoResearch Laboratories, Inc	
Peroxidase-conjugated affinipure goat anti-rabbit IgG (H + L)	111-035-003	WB	Jackson IrnrnunoReserch Laboratories, Inc	
**Donkey anti-goat HRP/** AB_631728	Sc-2020	WB	Kirkegaard & Perry Laboratories, Inc	

Supplementary Table 3. Primers for qRT-PCR used in this study

Name	Forward Primer	Reverse Primer
ACTIN	CCTGGCACCCAGCACAATG	GGGCCGGACTCGTCATACT
BCL2	ACTGGCTCTGTCTGAGTAAG	CCTGATGCTCTGGGTAAC
c-MYC	TCTGGATCACCTTCTGCTGG	AGGATAGTCCTTCCGAGTGG
CCND1 (Cyclin D1)	GGATGCTGGAGGTCTGCGA	TAGAGGCCACGAACATGCAAGT
STAT3	CAGGAGGGCAGTTTGAGTCC	CAAAGATAGCAGAAGTAGGAGA
MCL1	CGGTAATCGGACTCAACCTC	CCTCCTTCTCCGTAGCCAA
CD44	CCCATTCGACAACAGGGACA	TGGGGTGTGAGATTGGGTTG
ABCB1	GGCCTAATGCCGAACACATT	CAGCGTCTGGCCCTTCTTC
ABCC1	AACCTGGACCCATTCAGCC	GACTGGATGAGGTCGTCCGT

**Supplementary Table 4.** Signal Dual-Luciferase Reporter Assays used in the study				
**Pathway**	**Transcription Factor**	**Dual luciferase Cat. No.**	**MGFP Cat. No.**	Vendor QIAGEN **Note**
MAPK/JNK	AP-1	CCS-011L	CCS-011G	
Antioxidant Response	Nrf2 & Nrf1	CCS-5020L		
Hypoxia	HIF-1	CCS-007L		HRE
NFκB	NFκB	CCS-013L	CCS-013G	
STAT3	STAT3	CCS-9028		
PPAR	PPAR	CCS-3026L		PPRE
TGFβ	SMAD2/SMAD3/SMAD4	CCS-017L	CCS-017G	
Wnt	TCF/LEF	CCS-018L	CCS-018G	

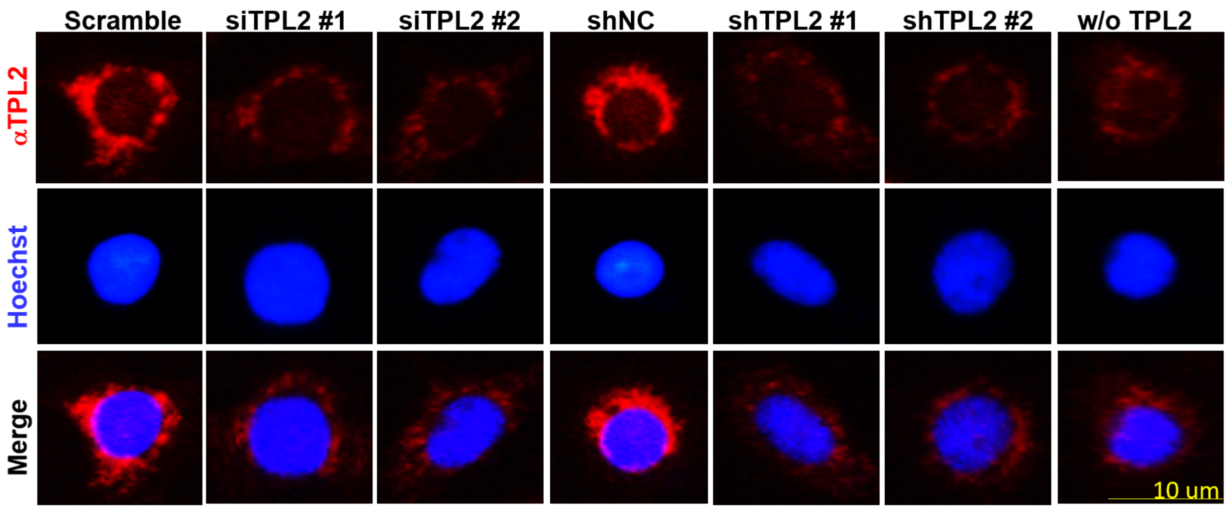

Supplementary Fig 1

Supplementary Fig. 1. Validation of TPL2 knockdown by siRNA and shRNA in AGS cells using immunofluorescence microscopy. AGS gastric cancer cells were transfected with either control or TPL2-targeting RNA interference reagents and analyzed by immunofluorescence to assess TPL2 protein expression. The experimental conditions were as follows: Scramble: cells transfected with a non-targeting scrambled siRNA control. siTPL2#1 and siTPL2#2: cells transfected with two independent TPL2-specific siRNAs. **shNC:** cells infected with a lentiviral vector expressing a non-targeting scrambled shRNA (short hairpin RNA) control. shTPL2#1 and shTPL2#2: cells infected with lentiviral vectors expressing two different TPL2-targeting shRNAs. w/o TPL2: negative control with no TPL2 staining (primary antibody omitted). At 24 hours post-transfection (siRNA) or post-infection (shRNA), cells were fixed and stained with an anti-TPL2 antibody (red) to assess protein levels. Nuclei were counterstained with Hoechst dye (blue). Effective TPL2 knockdown was observed in both siRNA- and shRNA-treated groups, with reduced TPL2 signal compared to controls, confirming the efficacy and specificity of the RNA interference reagents. Scale bar = 10 μm.

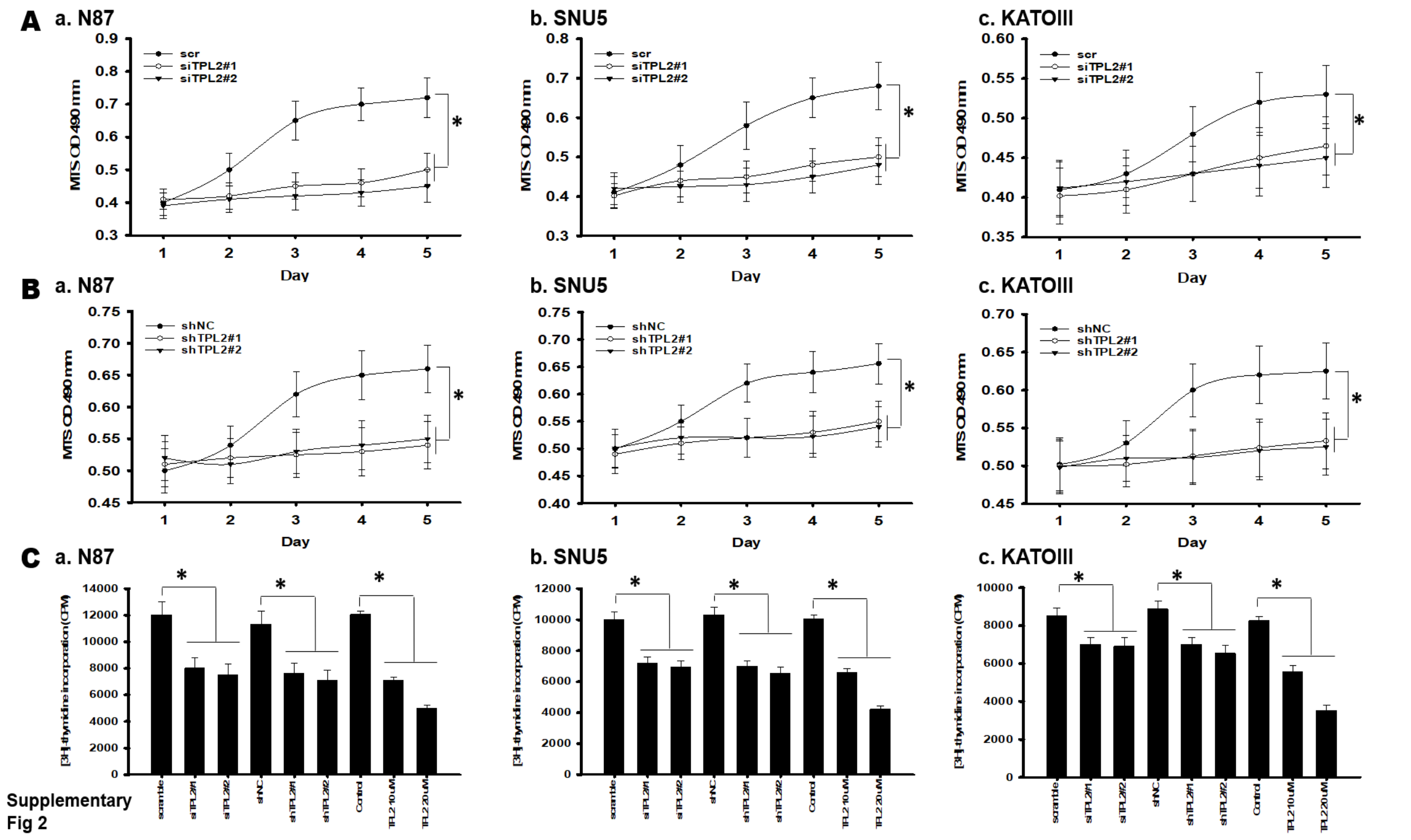

Supplementary Fig. 2. TPL2 downregulation suppresses gastric cancer (GC) cell proliferation in N87 (a), SNU (b), and KATOIII (c) cell lines.

2A. MTS assay analysis of TPL2 knockdown using scrambled siRNA (scr) and siTPL2 in (a) N87, (b) SNU and (c) KATOIII cell lines (n = 6). *p < 0.05.

2B. MTS assay analysis of TPL2 knockdown using a non-targeting shRNA control (shNC) and shTPL2 in (a) N87, (b) SNU and (c) KATOIII cell lines (n = 6). *p < 0.05.

2C. Thymidine incorporation assay was used to assess cell proliferation following TPL2 knockdown or treatment with a TPL2 inhibitor in (a) N87, (b) SNU and (c) KATOIII cell lines. Cell number was recorded (n = 6). *p < 0.05.

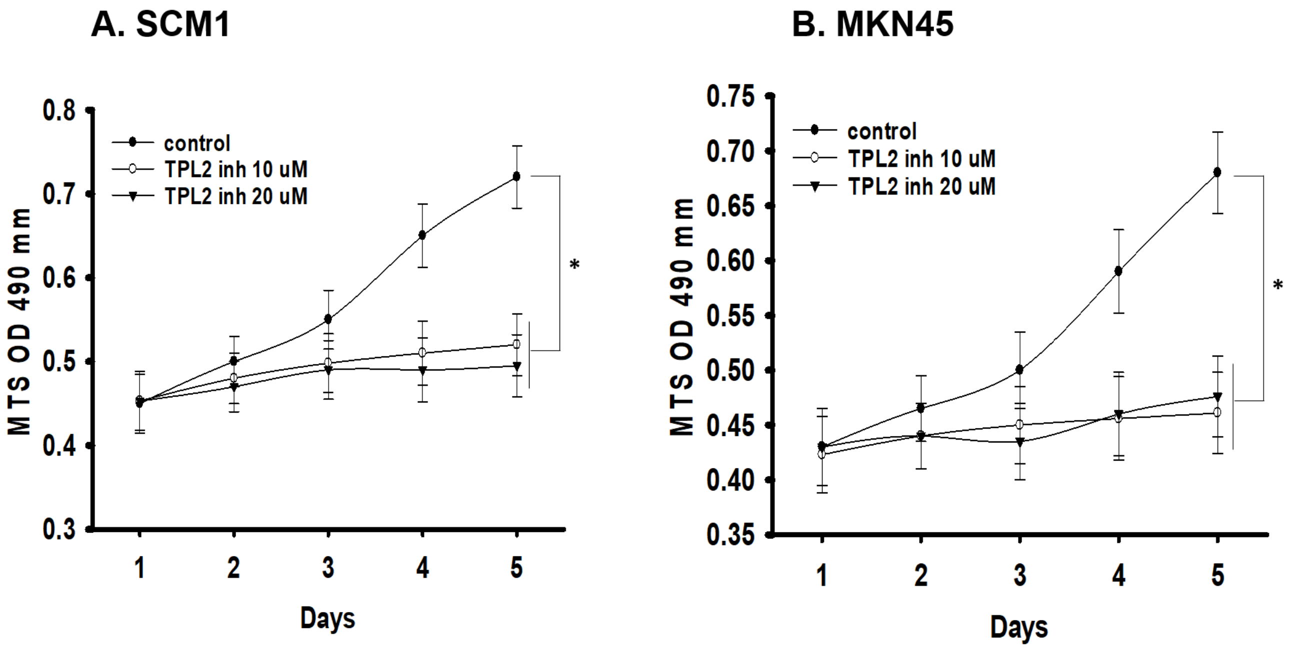

Supplementary Fig 3

Supplementary Fig. 3. Specific pharmacological inhibition of TPL2 suppressed gastric cancer cell proliferation in SCM1 (A) and MKN45 (B) cell lines. MTS assay analysis showed that treatment with a TPL2 inhibitor (10–20 μM) significantly reduced cell viability in both SCM1 and MKN45 cells (n = 6). *$p < 0.05$.

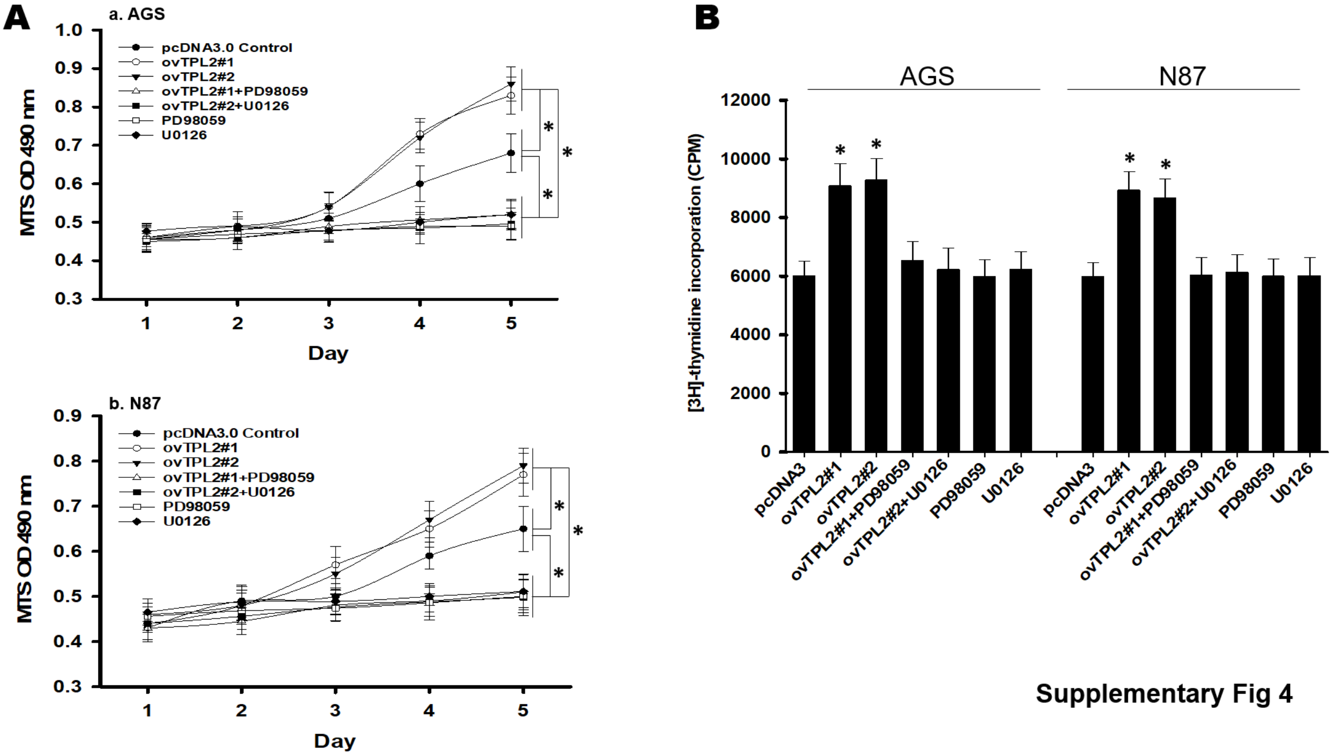

Supplementary Fig. 4. Specific pharmacological inhibition of MEK U0126 and PD98059 suppressed gastric cancer cell proliferation in AGS and N87 cell lines.

4A. MTS assay analysis showed that treatment with U0126 and PD98059, with or without TPL2 overexpression, significantly reduced cell viability in AGS (a) and N87 (b) gastric cancer cells. *$p < 0.05$.

4B. Thymidine incorporation assay was performed to assess cell proliferation following TPL2 overexpression or treatment with U0126 and PD98059 in AGS and N87 cells. Cell numbers were recorded ($n = 6$). *$p < 0.05$.

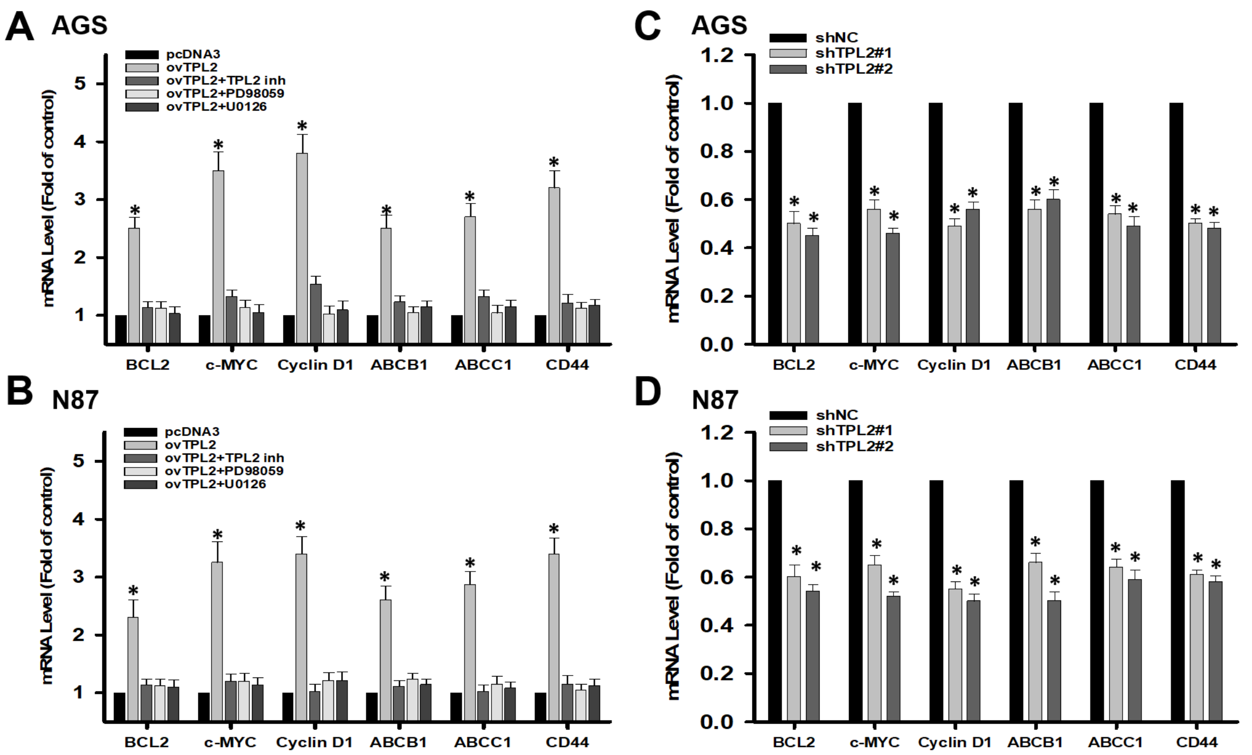

Supplementary Fig 5

Supplementary Fig. 5 Quantification of mRNA expression of proliferation and chemoresistance candidate genes regulated by TPL2/MEK signaling. Next, we analyzed the expression of downstream targets of proliferation gene by BCL2, c-MYC (MYC), Cyclin D1 (CCND1) and chemoresistance candidate ABCB1, ABCC1 and CD44 using qRT-PCR.

5A. Relative mRNA expression levels of BCL2, c-MYC, Cyclin D1, ABCB1, ABCC1, and CD44 in AGS/ovTPL2 cells treated with or without a TPL2 inhibitor or MEK inhibitors, as determined by RT-qPCR (n = 6). *$p < 0.05$.

5B. Relative mRNA expression levels of BCL2, c-MYC, Cyclin D1, ABCB1, ABCC1, and CD44 in N87/ovTPL2 cells treated with or without a TPL2 inhibitor or MEK inhibitors, as determined by RT-qPCR (n = 6). *$p < 0.05$.

5C. Relative mRNA expression levels of BCL2, c-MYC, Cyclin D1, ABCB1, ABCC1, and CD44 in AGS/shTPL2 cells, as determined by RT-qPCR (n = 6). *$p < 0.05$.

5D. Relative mRNA expression levels of BCL2, c-MYC, Cyclin D1, ABCB1, ABCC1, and CD44 in N87/shTPL2 cells, as determined by RT-qPCR (n = 6). *$p < 0.05$.

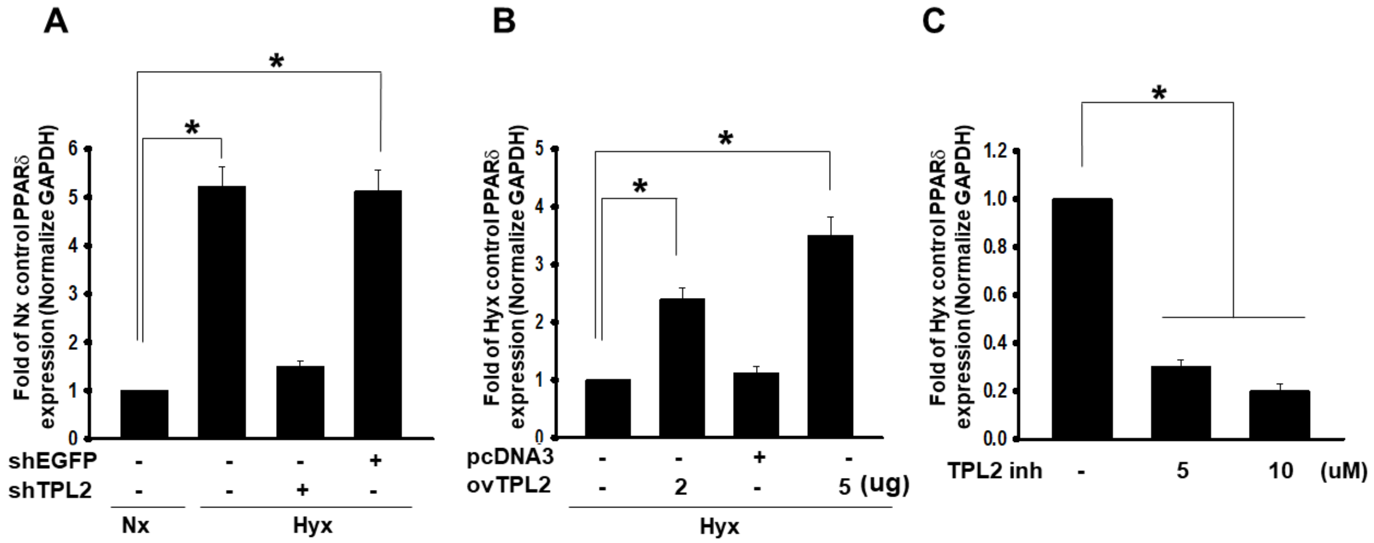

Supplementary Fig 6

Supplementary Fig. 6. Gastric cancer cells were transfected with shEGFP, shMAP3K8 (shTPL2), pcDNA3, or pDONR223-MAP3K8 (ovTPL2), or treated overnight with a TPL2 inhibitor, followed by hypoxia treatment. Image quantification was performed and compared to the indicated normoxia or hypoxia groups (n = 5). *$p < 0.05$.*

6A. Western blot analysis of PPARδ protein expression in cells transfected with shEGFP or shTPL2.

6B. Western blot analysis of PPARδ protein expression in hypoxic cells transfected with pcDNA3 or ovTPL2. Quantification of PPARδ protein levels is presented.

6C. Western blot analysis of PPARδ protein expression in hypoxic cells treated with a TPL2 inhibitor. All quantification values are presented as the mean ± SD from five independent biological replicates.

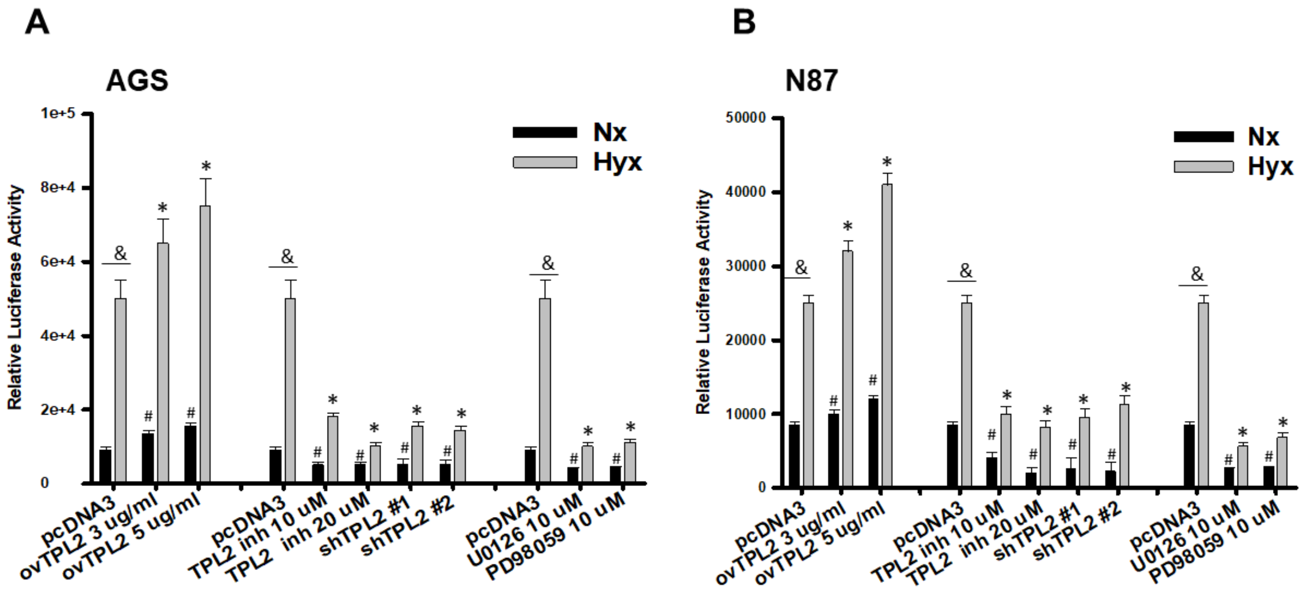

Supplementary Fig 7

Supplementary Fig. 7. TPL2 regulates PPARδ production in a kinase activity–dependent manner. Gastric cancer cells were transfected with pcDNA3, overexpressed TPL2 (ovTPL2), or shTPL2, and treated with or without a TPL2 inhibitor under normoxic or hypoxic conditions. PPAR response element (PPRE) luciferase activity was subsequently measured in AGS (A) or N87 (B) gastric cancer cells. &$p < 0.05$ vs. normoxic condition; #$p < 0.05$ vs. normoxic control; #$p < 0.05$ vs. hypoxic group, as indicated.

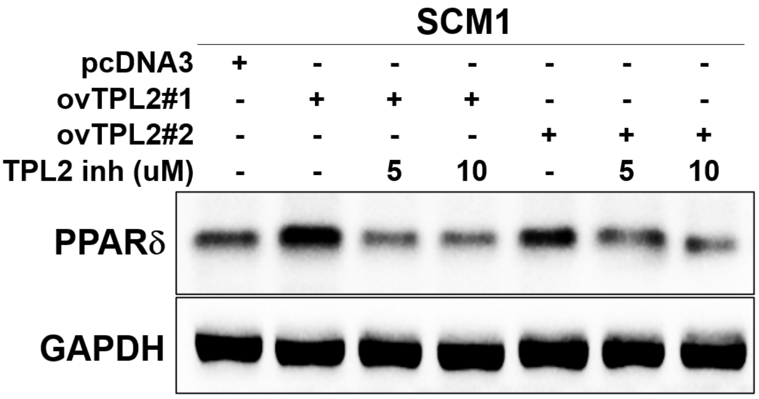

Supplementary Fig 8

Supplementary Fig. 8. TPL2 regulates PPARδ production in a kinase activity–dependent manner, as determined by Western blot analysis. SCM1 gastric cancer cells were transfected with pcDNA3 or overexpressed TPL2 (ovTPL2), and treated with or without a TPL2 inhibitor under hypoxic conditions. PPARδ protein levels were subsequently measured.

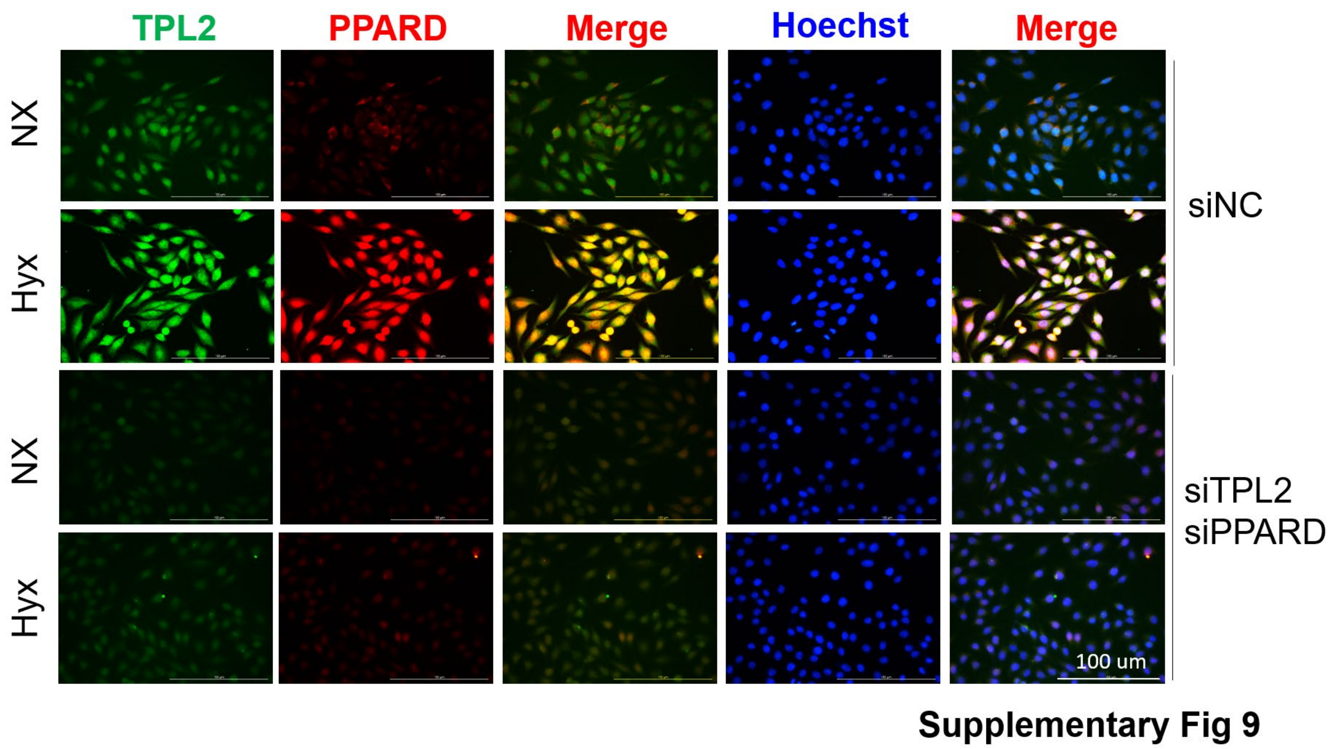

Supplementary Fig. 9. Evaluate the effects of gene silencing using image-based immunofluorescence analysis. The effects of gene silencing by siRNA, siRNA-TPL2, or siRNA-PPARδ were evaluated using image-based immunofluorescence analysis. TPL2 was detected with a primary antibody and visualized in green; PPARδ was detected with a primary antibody and visualized in red; nuclei were counterstained with Hoechst dye (blue). The images confirm successful knockdown of TPL2 in SCM1 cells and demonstrate specific staining of both TPL2 and PPARδ.

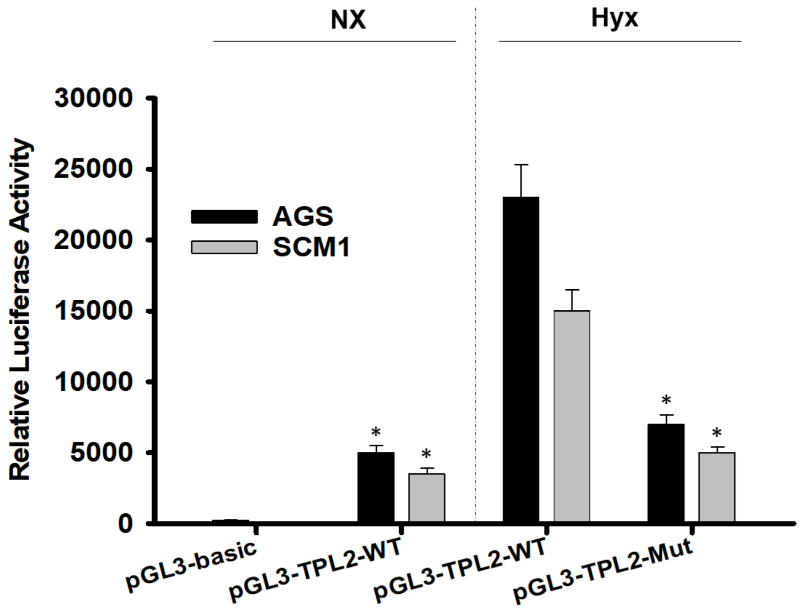

Supplementary Fig 10

Supplementary Fig. 10. Mutation in the PPRE element attenuates the effect of PPARδ on TPL2 promoter activity in AGS and SCM1 cells. Gastric cancer cell lines AGS and SCM1 were co-transfected with luciferase reporter constructs containing the TPL2 promoter region (pGL3-TPL2) or a version with a mutated PPRE site at position -370 (pGL3-TPL2 PPREmut). Luciferase activity was measured 24 hours post-transfection. Promoter fragments spanning from -1937 to -370 relative to the transcription start site (TSS) were utilized, along with a series of deletion constructs. Relative luciferase activity was determined by normalizing Firefly luciferase activity to Renilla luciferase activity. All experiments were performed in triplicate and independently repeated at least three times. Data are presented as mean ± SEM. $*p < 0.01$ compared to the normoxia or hypoxia control group, as indicated.

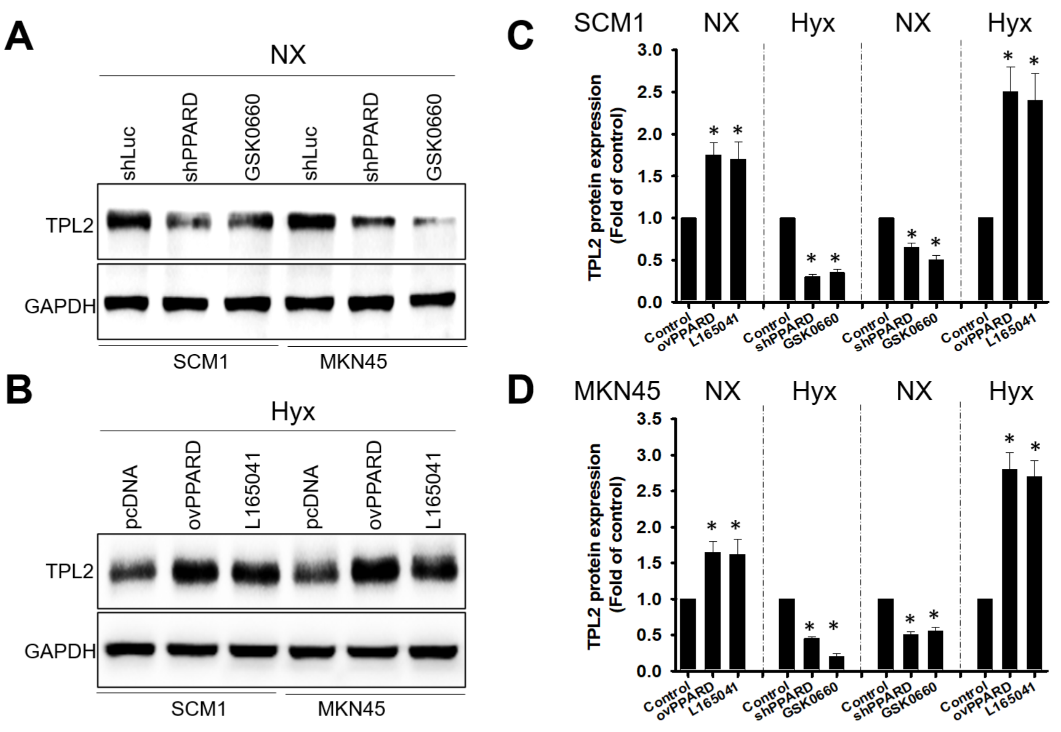

Supplementary Fig 11

Supplementary Fig. 11. PPARδ regulates TPL2 protein expression in SCM1 and MKN45 cells. TPL2 expression was significantly modulated by PPARδ. SCM1 and MKN45 cells were transfected with either PPARδ overexpression (ovPPARδ) or shRNA-mediated knockdown (shPPARδ) plasmids. Additionally, cells were treated with L-165,041, a potent PPARδ agonist, or GSK0660, a selective PPARδ antagonist, to further evaluate the regulatory effects of PPARδ on TPL2.

11A. Under normoxic conditions, western blot analysis was performed to assess TPL2 protein levels in cells transfected with shLuc or shPPARδ, with or without treatment with GSK0660.

11B. Under hypoxic conditions, western blot analysis was conducted to evaluate TPL2 expression in cells transfected with pcDNA3 or ovPPARδ, with or without treatment with L-165,041.

11C-11D. Quantification of the results is based on at least five independent experiments. Data are presented as mean ± SEM. **p* < 0.01 compared to the normoxia or hypoxia control group in SCM1 and MKN45 cells, as indicated.

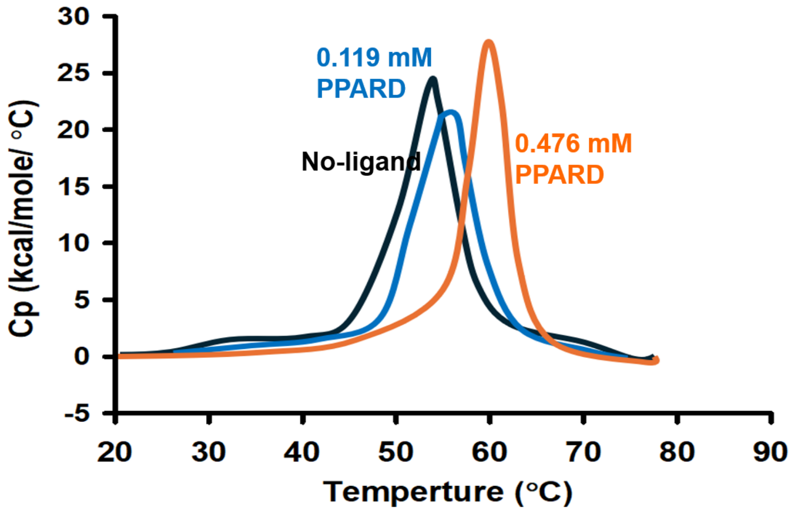

Supplementary Fig 12

Supplementary Fig. 12. TPL2–PPARδ protein–protein interactions by Differential scanning calorimetry (DSC). Differential scanning calorimetry (DSC) analysis of TPL2–PPARδ protein–protein interactions. This protocol aimed to assess the thermal stability of TPL2 in the presence or absence of recombinant human PPARδ using DSC. TPL2 (14.2 μM) was prepared in 50 mM HEPES, 0.1 M KCl, and 10 mM β-mercaptoethanol at pH 7.2. Samples were subjected to thermal denaturation by heating from 20 °C to 80 °C at a rate of 90 °C per hour. Thermal profiles were recorded under the following conditions: black lines, no ligand; blue lines, 0.119 mM PPARδ; red lines, 0.476 mM PPARδ. Similar thermal contours were observed when the experiment was performed in an alternative buffer containing 50 mM potassium phosphate (pH 7.2) and 1 mM dithiothreitol. All results are representative of at least five independent experiments and are presented accordingly.

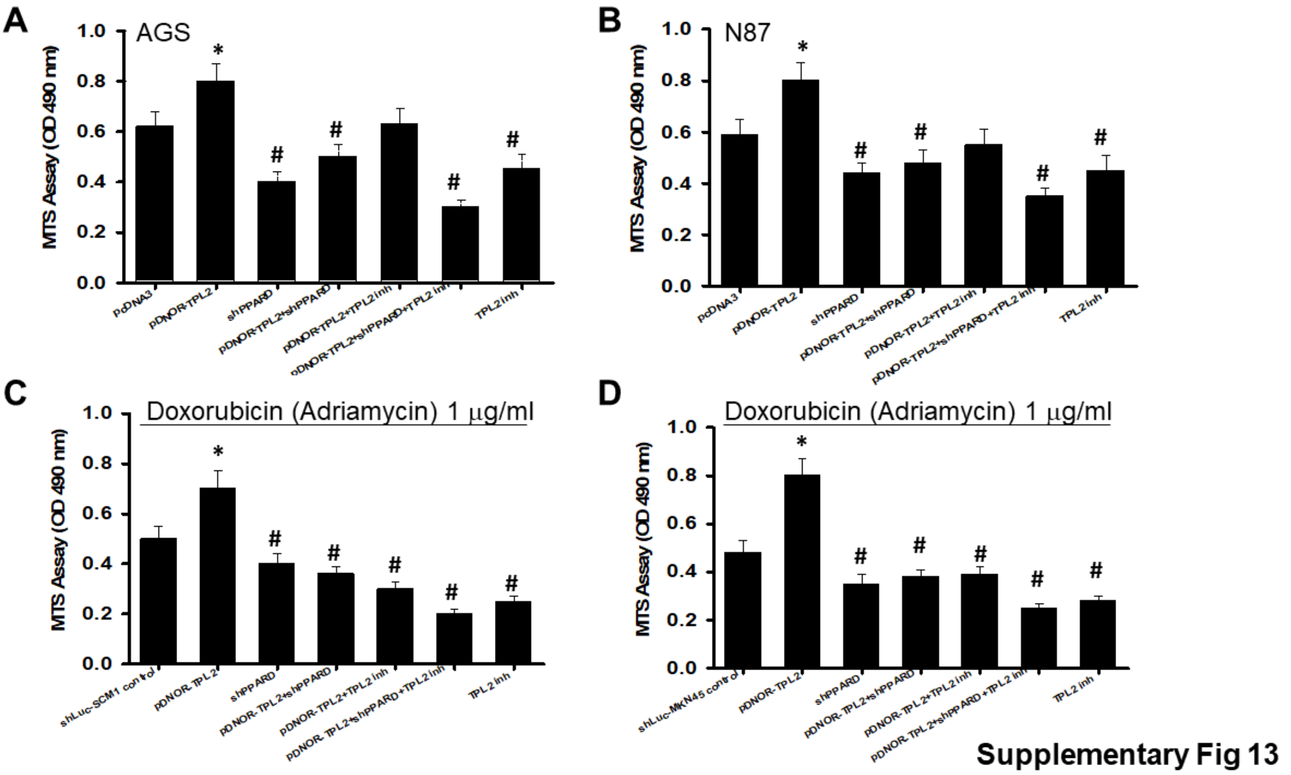

Supplementary Fig. 13. TPL2 kinase activity is required for the functional interaction between TPL2 and PPARδ in regulating cell proliferation and Doxorubicin resistance. AGS and N87 cells were transfected with either a TPL2 overexpression plasmid (ovTPL2; pDONR-TPL2) or an shRNA plasmid targeting PPARδ (shPPARδ). Additionally, cells were treated with a TPL2 inhibitor to assess the contribution of TPL2 kinase activity to the regulatory effects of PPARδ and TPL2.

13A. Cell viability was assessed by MTS assay in AGS/TPL2 cells with or without PPARδ knockdown and TPL2 inhibitor treatment.

13B. Cell viability was assessed by MTS assay in N87/TPL2 cells with or without PPARδ knockdown and TPL2 inhibitor treatment.

13C. Cell viability was measured by MTS assay in SCM1 cells treated with increasing concentrations of Doxorubicin, as indicated.

13D. Cell viability was measured by MTS assay in MKN45 cells treated with increasing concentrations of Doxorubicin, as indicated.

Data are presented as mean ± SEM from at least three independent experiments.

#$p < 0.05$ compared to the control group. *$p < 0.05$ compared to the respective treatment control group.

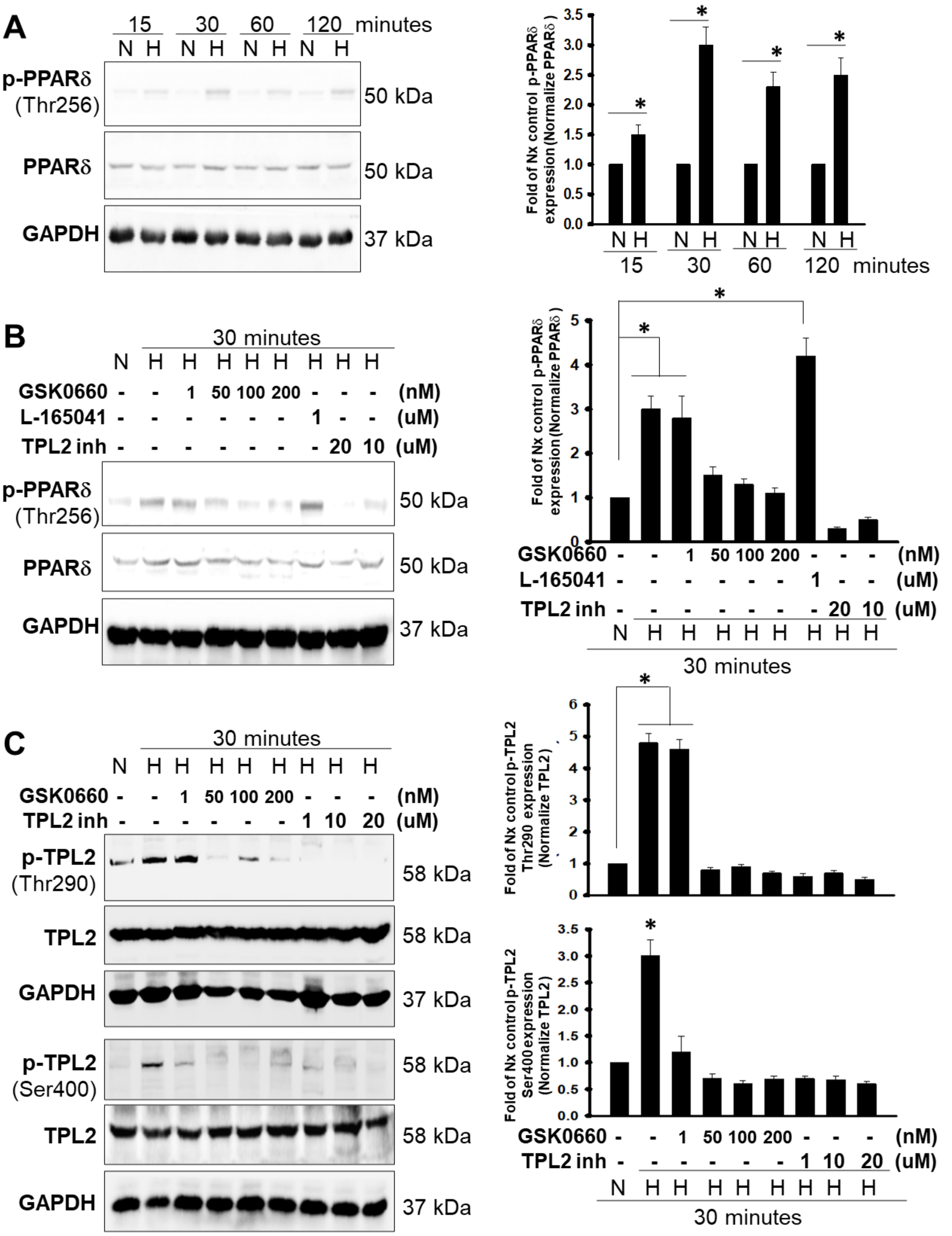

Supplementary Fig 14

Supplementary Fig. 14. Hypoxia-induced phosphorylation of PPARD and activation of TPL2.

14A. SCM1 cells were incubated for15~120 minutes under normoxic (21% O2) or hypoxic (1% O2 and 5% O2) conditions. Western blot detection phosphorylation of PPARD (Thr256), PPARD and internal control GAPDH protein production.

14-B-14C. Pretreatment PPARD antagonist (GSK0660; 1-200 nM) agonist (L-165041; 1uM) and TPL2 inhibitors (10-20 uM) following hypoxic exposure. Primary antibody detection for p-PPARD (Thr256), PPARD, p-TPL2 (Thr290), p-TPL2 (Ser400), TPL2 and internal control GAPDH protein production. Whole-cell lysates were fractionated on 8% polyacrylamide gels with SDS-PAGE and immunoblotted with an anti-HIF-1α, anti-HIF-2α, or anti-GAPDH antibody (as the loading control).

Right panel shown the quantitation analysis. Similar western blots were obtained in six independent experiments. * $P<0.05$ compared with control group.

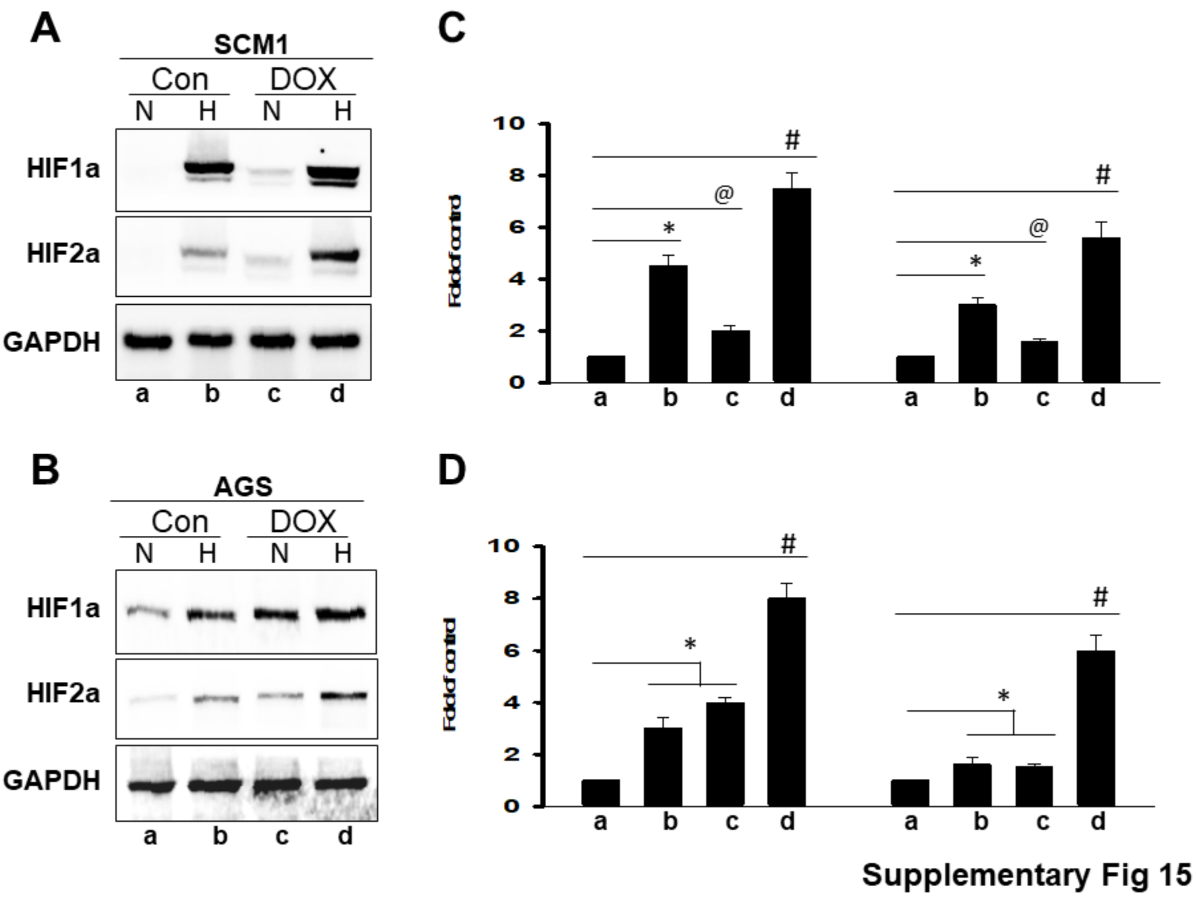

Supplementary Fig. 15 The increase in HIF protein expression by doxorubicin under hypoxia condition.

15A, 15B. SCM1 and AGS cells were incubated for 24 h with or without doxorubicin (DOX 50 nM) under normoxic (21% O2) or hypoxic (1% O2 and 5% O2) conditions. Whole-cell lysates were fractionated on 8% polyacrylamide gels with SDS-PAGE and immunoblotted with an anti-HIF-1α, anti-HIF-2α, or anti-GAPDH antibody (as the loading control).

15C, 15D. The ratios of HIF/β-actin are shown as fold changes. Quantification values are expressed as the means ± SD of six independent biological replicates. *or @ or # $P<0.05$ compared with control group.

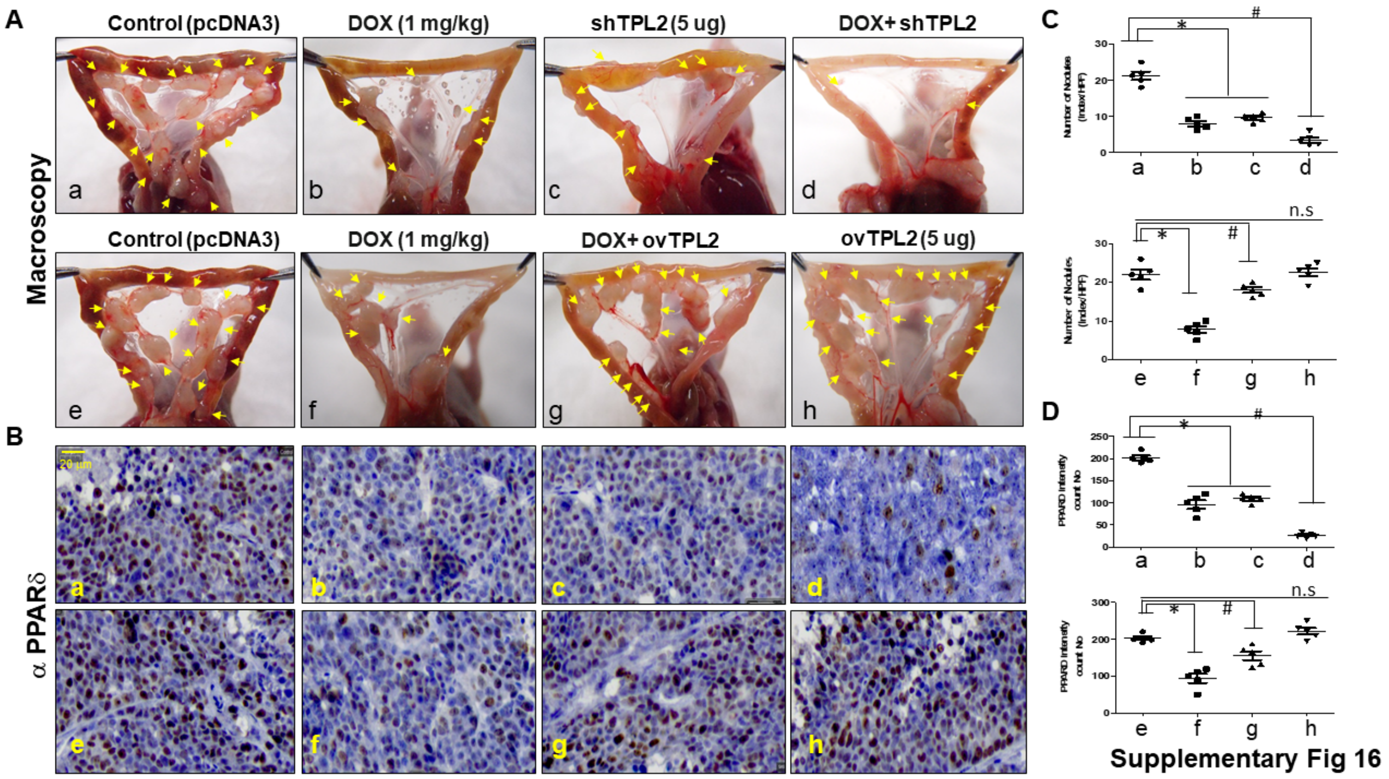

Supplementary Fig. 16. TPL2 regulated doxorubicin-treatment in peritoneal dissemination animal model.

16A, Nude mice inoculated intraperitoneal (i.p.) with MKN45 cells (pcDNA3), transfected with shTPL2 (5 µg) or were ovTPL2 (5 µg) treated with DOX (1mg/kg) for 4 weeks. At the end of the treatment period, mice were sacrificed, macroscopic image capture and tumor specimens harvested. The tumor number was measured by macroscopic image/HPF. Mean tumor volume for each treatment group is indicated. Results are represented as 5 mice per group (n = 5). * Statistically significant decrease compared to control (p<0.05) and # lower than DOX group (p<0.05). n.s. means not significant, p > 0.05

16B, Tumor specimens were subjected to immunohistochemical staining for PPARδ. Images were captured at 400× magnification. The Scale bar represents 20 µm. Mean density of each group (Integrated optical density/Area) was presented (n = 5, * p < 0.05; n.s. means not significant, p > 0.05).

16C-16D Quantification values are expressed as the means ± SD of five independent biological replicates.

*or # P<0.05 compared with control group.

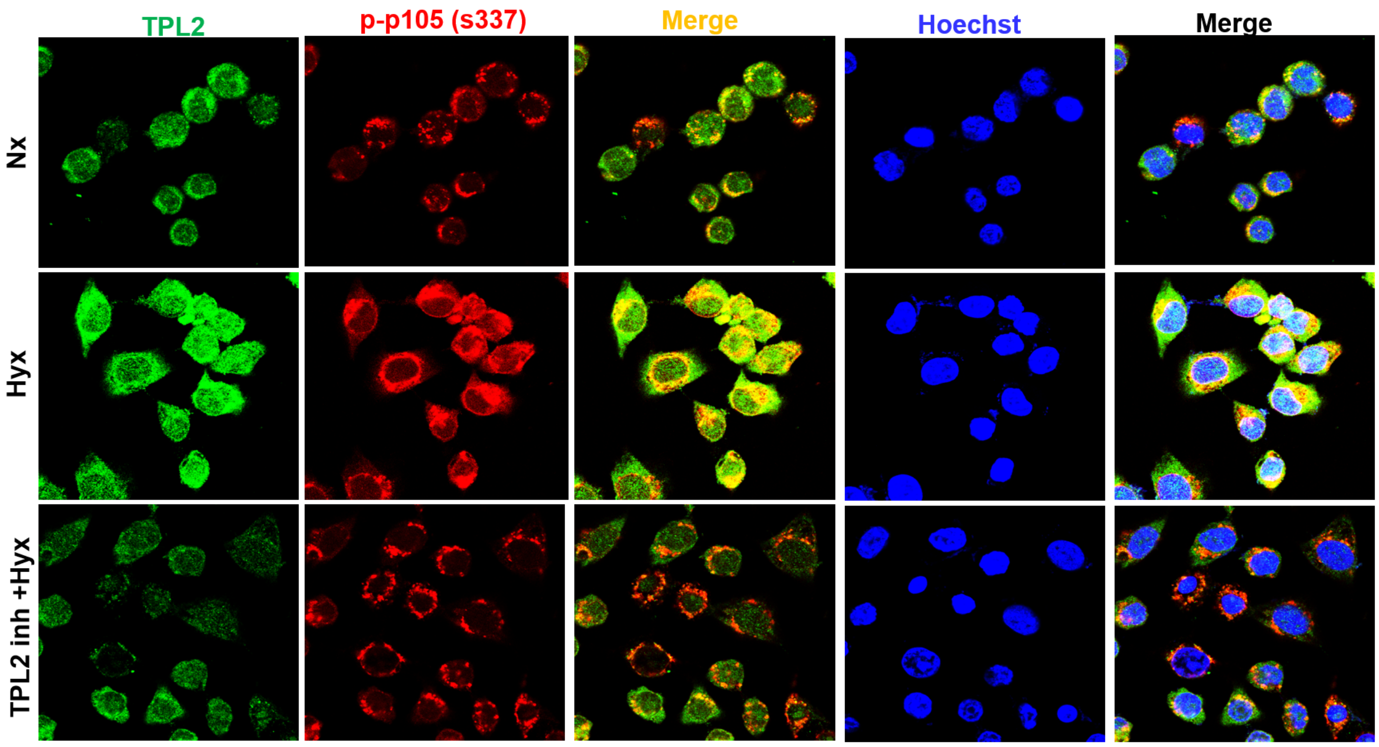

Supplementary Fig 17

Supplementary Fig. 17. TPL2 Inhibitor Disrupts TPL2–NF-κB p105 Interaction in Gastric Cancer Cells Under Hypoxia. SCM1 gastric cancer cells were pre-treated with a specific pharmacological TPL2 inhibitor under hypoxic conditions for 8 hours to assess the effect on TPL2 interaction with the NF-κB p105 subunit. Colocalization was examined by immunofluorescence staining. TPL2 was labeled in green, phospho-NF-κB1 (Ser337) in red, and nuclei were counterstained with Hoechst dye (blue). Under resting conditions, TPL2 forms a stable complex with the NF-κB precursor protein p105, sequestering it in an inactive state. Hypoxic stimulation leads to phosphorylation of p105, promoting its ubiquitination and proteasomal degradation. This degradation releases TPL2, enabling its kinase activity and activation of downstream signaling pathways involved in cellular responses. The immunofluorescence images confirm effective TPL2 inhibition in SCM1 cells and demonstrate specific staining and disrupted colocalization of TPL2 and p105 following inhibitor treatment.

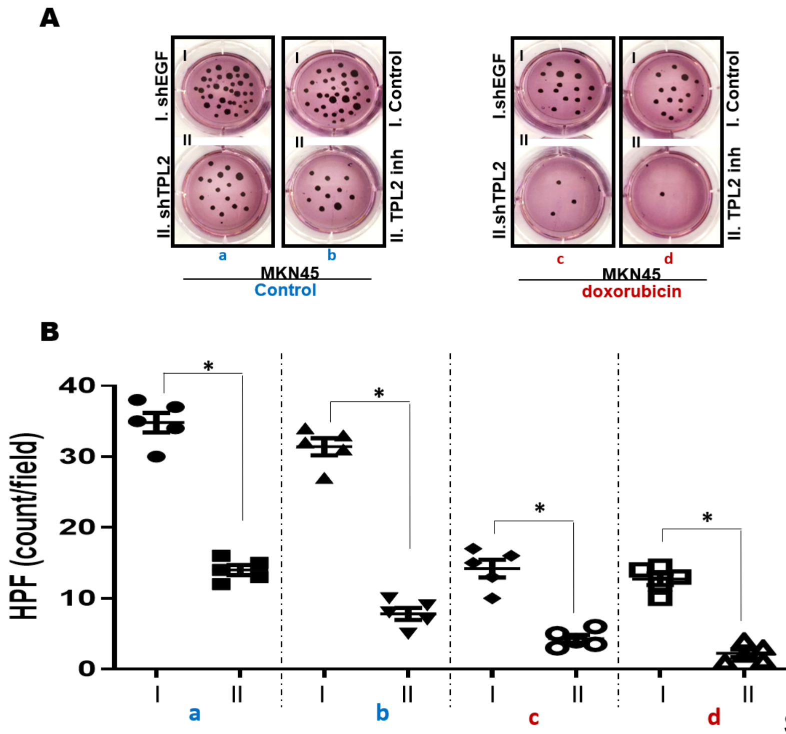

Supplementary Fig 18

Supplementary Fig. 18. Colony formation assay of TPL2 knockdown in MKN45 cells with or without chemotherapy doxorubicin treatment (100 nM).

A. “I” and “II” denote the subgroups as follows:

a. Inoculation with gastric cancer MKN45 cells transfected with either group I: shEGF or group II: shTPL2;

b. Inoculation with gastric cancer MKN45 cells treated with either group I: control or group II: TPL2 inhibitor (inh);

c. Inoculation with gastric cancer MKN45 cells transfected with either Group I: shEGF or Group II: shTPL2, followed by doxorubicin treatment (100 nM).

d. Inoculation with gastric cancer MKN45 cells treated with either Group I: control or Group II: TPL2 inhibitor (inh), followed by doxorubicin treatment (100 nM). Subgroups a, b, c, and d in upper panel were quantified and presented in in lower panel.

B. Quantification in colony formation assay from upper panel was conducted for TPL2 knockdown in MKN45 cells, with or without doxorubicin treatment (100 nM) (n = 3). Statistical significance is indicated as *$p < 0.05$.

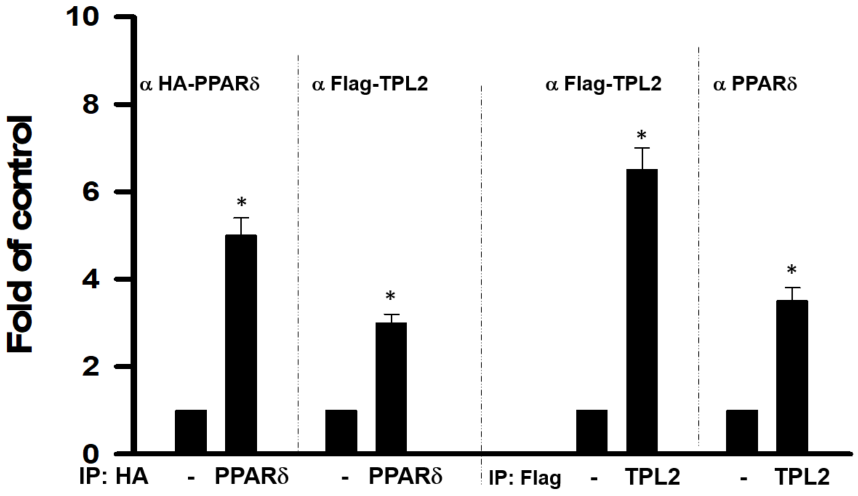

Supplementary Fig 19

Supplementary Fig. 19. Quantification of co-immunoprecipitation from Fig. 6B–6C demonstrates interaction between PPARδ and TPL2. Cells were co-transfected with plasmids encoding HA-tagged PPARδ or Flag-tagged TPL2 as indicated. Whole-cell lysates were subjected to immunoprecipitation (IP) using anti-HA or anti-Flag antibodies, followed by immunoblotting with the indicated antibodies. Quantification of the immunoprecipitated proteins is shown, with bars representing the fold enrichment relative to control (set to 1), normalized for input protein levels. A significant increase in fold enrichment indicates a specific interaction between PPARδ and TPL2. Data are presented as mean ± SEM. $p < 0.05$ compared to control.

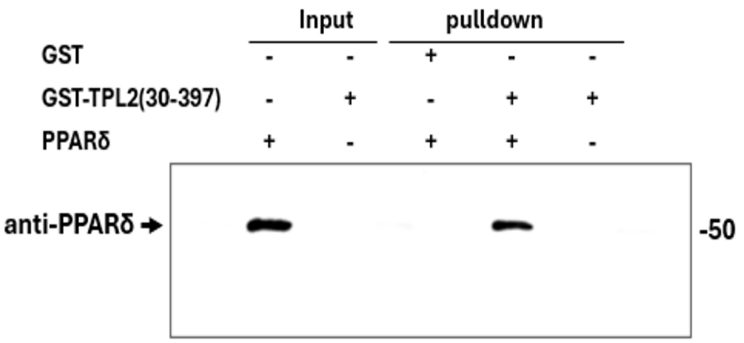

Supplementary Fig 20

Supplementary Fig. 20. TPL2–PPARδ protein–protein interactions by Pulldown assay. GST pulldown assay confirms interaction between GST-TPL2 (aa 30–397) and PPARδ. Pulldown assay was performed using GST-tagged TPL2 (amino acids 30–397) and full-length PPARδ. GST or GST-TPL2(30–397) was incubated with PPARδ, followed by glutathione sepharose pulldown and Western blotting with anti-PPARδ antibody. Input lanes show the presence of recombinant PPARδ and GST-TPL2. PPARδ was specifically detected in the GST-TPL2 pulldown lane (Lane 4), but not in the GST-only control (Lane 3) or in the absence of PPARδ (Lane 5), confirming specific binding between PPARδ and the truncated TPL2 construct used in the DSC.

Supplementary Materials:

Differential Scanning Calorimetry (DSC)

To perform Differential Scanning Calorimetry (DSC) on recombinant human PPARD (Peroxisome Proliferator Activated Receptor Delta; Catalogue No: 102080, aa 2-441, BPS Bioscience, Inc.; Catalog No: abx068495, aa 260-426, Abbex , Inc.) and MAP3K8/Tpl2/COT (Catalog No: 4586-KS, aa 30-397, R&D Systems, Inc.), purified protein samples must first be buffer-exchanged into a DSC-compatible buffer such as 20 mM HEPES, 150 mM NaCl, pH 7.4 to minimize baseline noise and ensure thermal stability. The protein concentrations should be adjusted to approximately 0.1–1.0 mg/mL, depending on instrument sensitivity. Samples are thoroughly degassed to prevent bubble formation, then loaded into the sample cell of a DSC instrument, with matched buffer in the reference cell. A temperature scan, typically ranging from 10 °C to 90 °C at a rate of 1 °C/min, is performed to detect thermal transitions. The resulting thermograms allow assessment of protein stability and folding characteristics by identifying melting temperatures (Tm) and calorimetric enthalpy (ΔH_cal). Proper cleaning and equilibration of the instrument between runs are essential for reliable, reproducible data.

Plasmid Constructs

Human proximal TPL2 promoter region (from -1937 to -370 relative to the transcription start site (TSS) was PCR amplified from the human genomic DNA with Q5® High Fidelity DNA Polymerase (New England Biolabs, Ipswich, MA, USA) using TPL2 f and TPL2 rev primers and cloned through BglII and HindIII sites into the pGL3 basic vector (Promega, Madison, WI, USA). Cloning resulted in the pGL3-TPL2 plasmid vector that was sequence verified. Mutations in the PPARD region of TPL2 promoter were introduced with the Quick-Change II Site-Directed Muta genesis kits (Stratagene, San Diego, CA, USA). All the plasmid vectors were sequence verified by Sanger sequencing (GATC, Konstanz, Germany). Luciferase activity was measured 24 hours post-transfection, with Firefly luciferase activity normalized to Renilla luciferase to control for transfection efficiency.

GST pull-down assay. GST alone or GST-tagged TPL2 (amino acids 30–397) was expressed in *E. coli* and

immobilized on glutathione-Sepharose beads. The beads were incubated with lysates containing overexpressed PPARδ protein. After extensive washing, bound proteins were eluted and analyzed by SDS-PAGE followed by immunoblotting with anti-PPARδ antibody. PPARδ was specifically pulled down by GST-TPL2(30–397) but not by GST alone, indicating a direct interaction between PPARδ and the N-terminal region of TPL2. Input represents 5% of total lysate used in the binding reaction.
